# Supplementary material for: Fibrolytic vaccination against ADAM12 reduces desmoplasia in preclinical pancreatic adenocarcinomas
Source: EMBO Mol Med. 2024 Oct 30;16(12):3033–56. doi: 10.1038/s44321-024-00157-4 (PMC11628623; doi:10.1038/s44321-024-00157-4)
Supplement: Supplementary file 1 — Appendix [file 44321_2024_157_MOESM1_ESM.pdf]

Table of Contents

Appendix Figure S1……………………………………………………..………………2

Appendix Figure S2…………………………………………………………...…………4

Appendix Figure S1

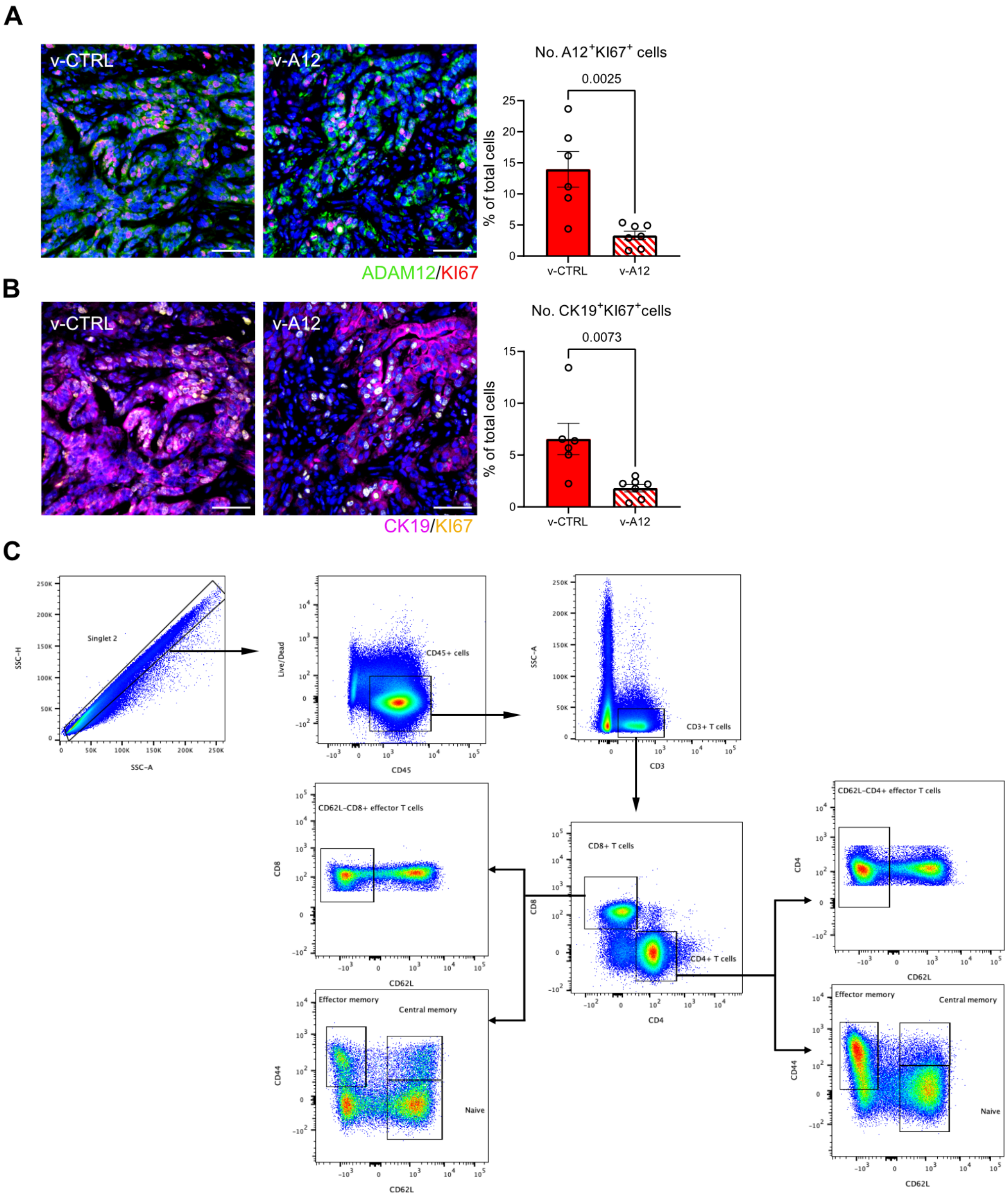

Appendix Figure S1. Prophylactic ADAM12 vaccination limited cell proliferation on subcutaneous KP2 PDAC tumor.

(A) Representative images (left) and quantitative analysis of proliferating ($KI67^+$) $ADAM12^+$ cells on PDAC tumors from v-CTRL and v-A12. (n= 6 mice in v-CTRL, n=7 mice in v-A12. Data are presented as mean ± SEM. Statistical test: unpaired two-tailed Student's t-test. Scale bar 50 μm)

(B) Representative images (left) and quantitative analysis of proliferating ($KI67^+$) tumor ($CK19^+$) cells on PDAC tumors from v-CTRL and v-A12 mice. (n=6 mice in v-CTRL, n=7 mice in v-A12. Data are presented as mean ± SEM. Statistical test: unpaired two-tailed Student's t-test. Scale bar 50 μm)

(C) Flow cytometry gating strategy of naïve, $CD62L^-$ effector $CD4^+$ T cells and $CD8^+$ T cells. $CD4^+$ T cells were identified as $CD45^+CD3^+CD4^+$ cells, and $CD8^+$ T cells as $CD45^+CD3^+$ $CD8^+$ cells. For naïve $CD4^+$ and $CD8^+$ T cells, they were defined as $CD62L^+CD44^-$ $CD4^+$ T cells or $CD62L^+CD44^-CD8^+$ T cells. For effector $CD4^+$ and $CD8^+$ T cells, they were defined as $CD62L^-CD4^+$ T cells and $CD62L^-CD8^+$ T cells, respectively.

Appendix Figure S2

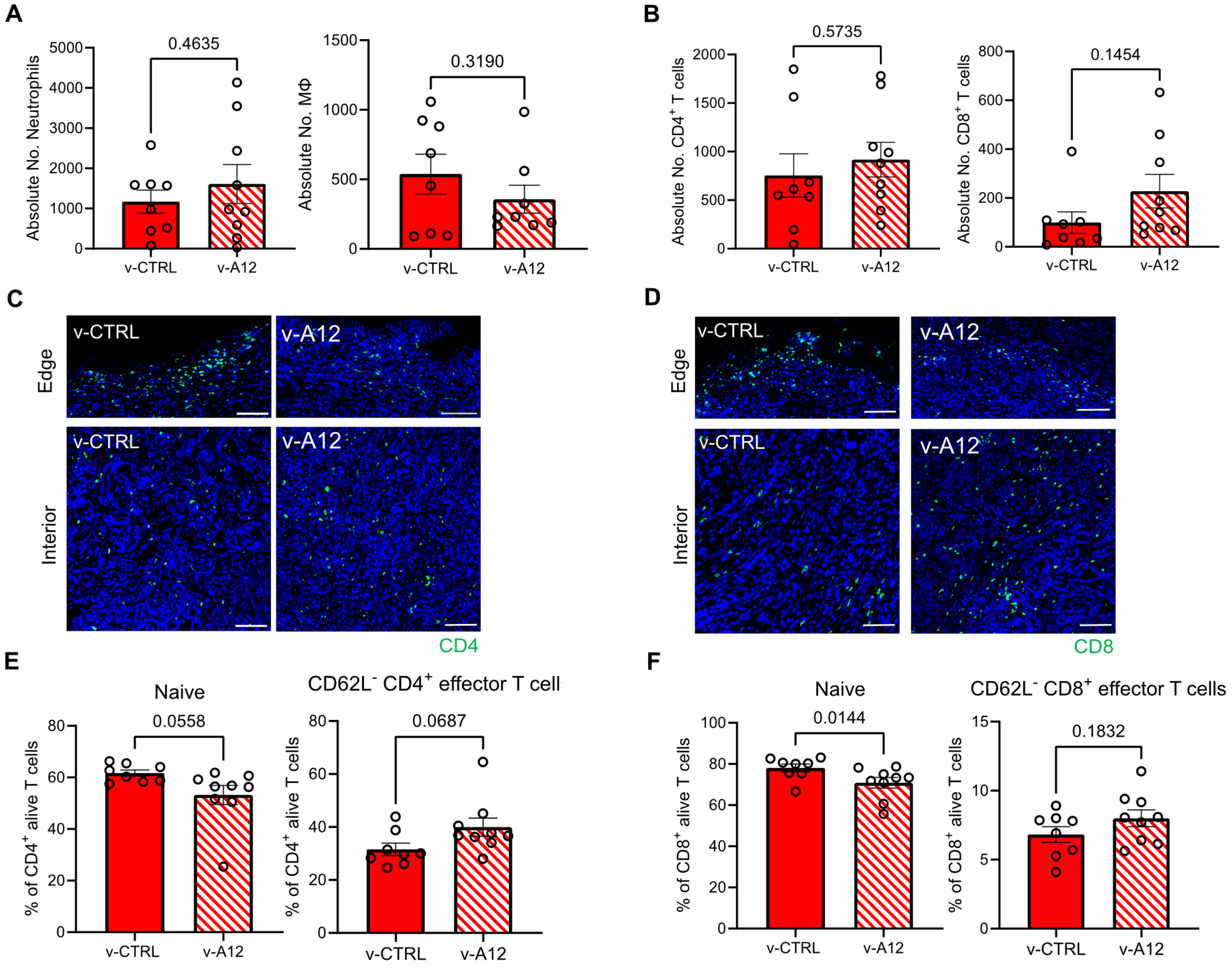

Appendix Figure S2. FACS analysis of spleens and PDAC tumors from mice with therapeutic ADAM12 vaccination.

(A) Quantitative analysis of the absolute number of neutrophils (left) and macrophages (right) within PDAC tumor tissues from control-vaccinated mice (v-CTRL) and ADAM12-vaccinated mice (v-A12). (For neutrophils, n=8 mice in v-CTRL, n=9 mice in v-A12; for macrophages, n=8 mice in v-CTRL, n=8 mice in v-A12. Data are presented as mean ± SEM. Statistical test: unpaired two-tailed Student's t-test.)

(B) Quantitative analysis of the absolute number of $CD4^+$ T cells (left) and $CD8^+$ T cells (right) within PDAC tumor tissues from control-vaccinated mice (v-CTRL) and ADAM12-vaccinated mice (v-A12). (n=8 mice in v-CTRL, n=9 mice in v-A12)

(C) Representative immunofluorescence staining images of $CD4^+$ T cells at the edge of the tumor tissue (upper) and $CD4^+$ T cells in the tumor tissue (bottom). (Scale bar 100 μm)

(D) Representative immunofluorescence staining images of $CD8^+$ T cells at the edge of the tumor tissue (upper) and $CD8^+$ T cells in the tumor tissue (bottom). (Scale bar 100 μm)

(E) Quantitative analysis via flow cytometry of naïve $CD4^+$ T cells (left) and effector/memory $CD4^+$ T cells (right) within splenic $CD4^+$ T cells from PDAC tumor-bearing mice treated with control vaccine (v-CTRL, n=8) and ADAM12 vaccine (v-A12, n=9). (n=8 mice in v-CTRL, n=9 mice in v-A12. Data are presented as mean ± SEM. Statistical test: unpaired two-tailed Student's t-test.)

(F) Quantitative analysis via flow cytometry of naïve $CD8^+$ T cells (left) and effector/memory $CD8^+$ T cells (right) within splenic $CD8^+$ T cells from PDAC tumor-bearing mice therapeutically treated with control vaccine (v-CTRL) and ADAM12 vaccine (v-A12). (n=8 mice in v-CTRL, n=9 mice in v-A12. Data are presented as mean ± SEM. Statistical test: unpaired two-tailed Student's t-test.)
