# Supplementary figures and images for: Fibrolytic vaccination against ADAM12 reduces desmoplasia in preclinical pancreatic adenocarcinomas
Source: EMBO Mol Med. 2024 Oct 30;16(12):3033–56. doi: 10.1038/s44321-024-00157-4 (PMC11628623; doi:10.1038/s44321-024-00157-4)

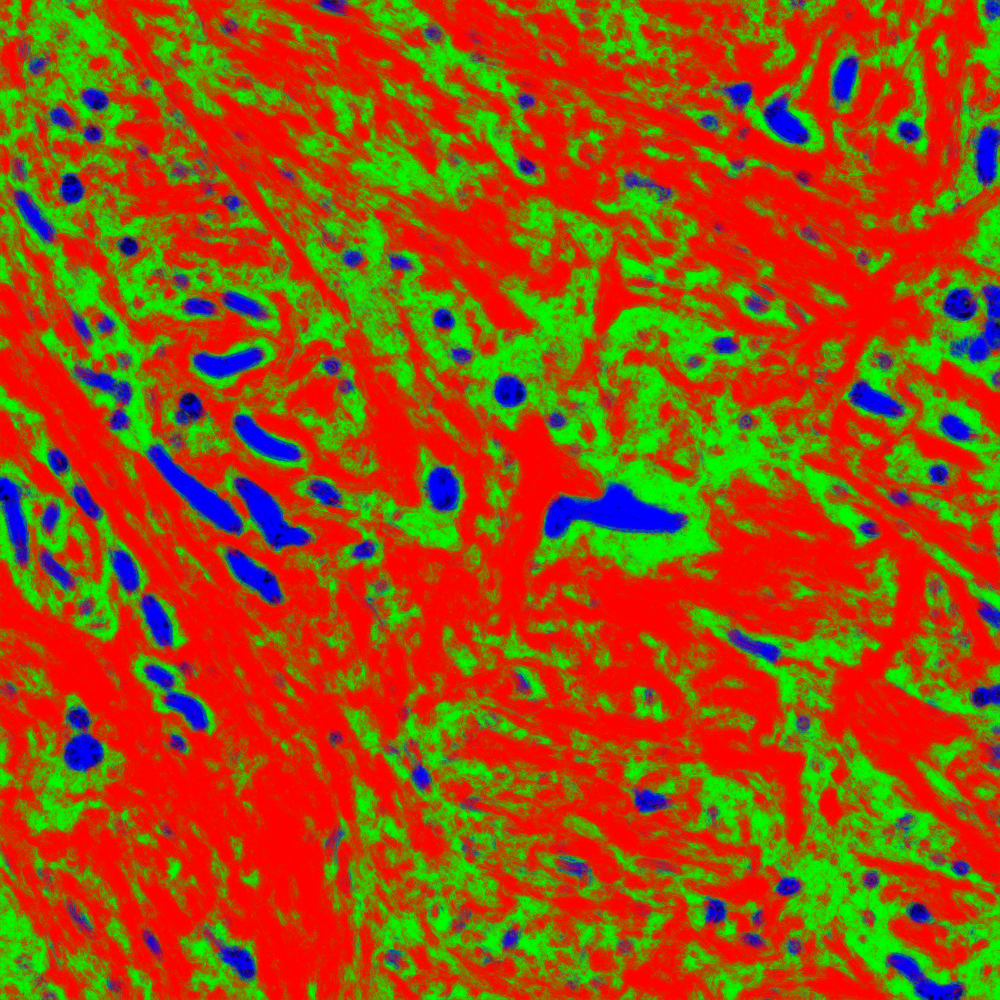

Supplement: Supplementary file 3 — Source data Fig. 1 [file 44321_2024_157_MOESM3_ESM.zip › Figure 1/1F tubular area/v-CTRL.tif]

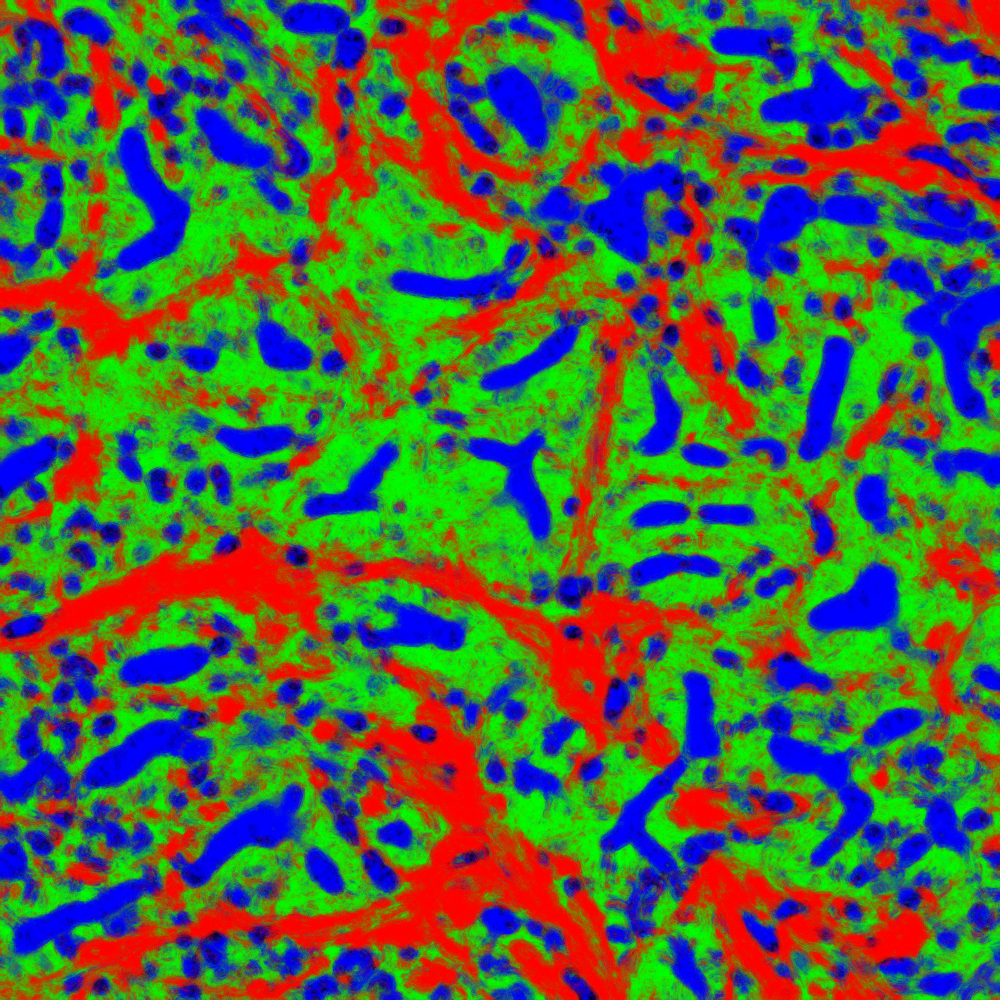

Supplement: Supplementary file 3 — Source data Fig. 1 [file 44321_2024_157_MOESM3_ESM.zip › Figure 1/1F tubular area/v-A12.tif]

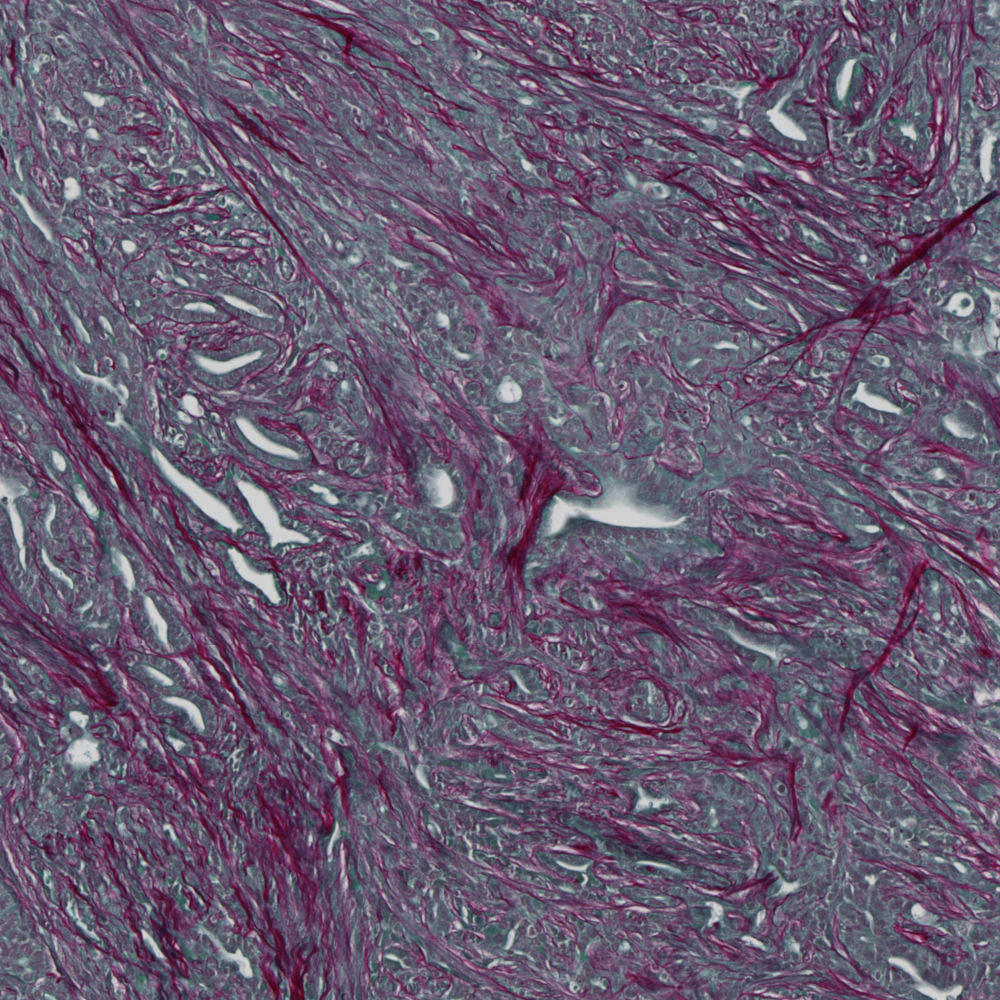

Supplement: Supplementary file 3 — Source data Fig. 1 [file 44321_2024_157_MOESM3_ESM.zip › Figure 1/1E collagen deposition/v-CTRL.tif]

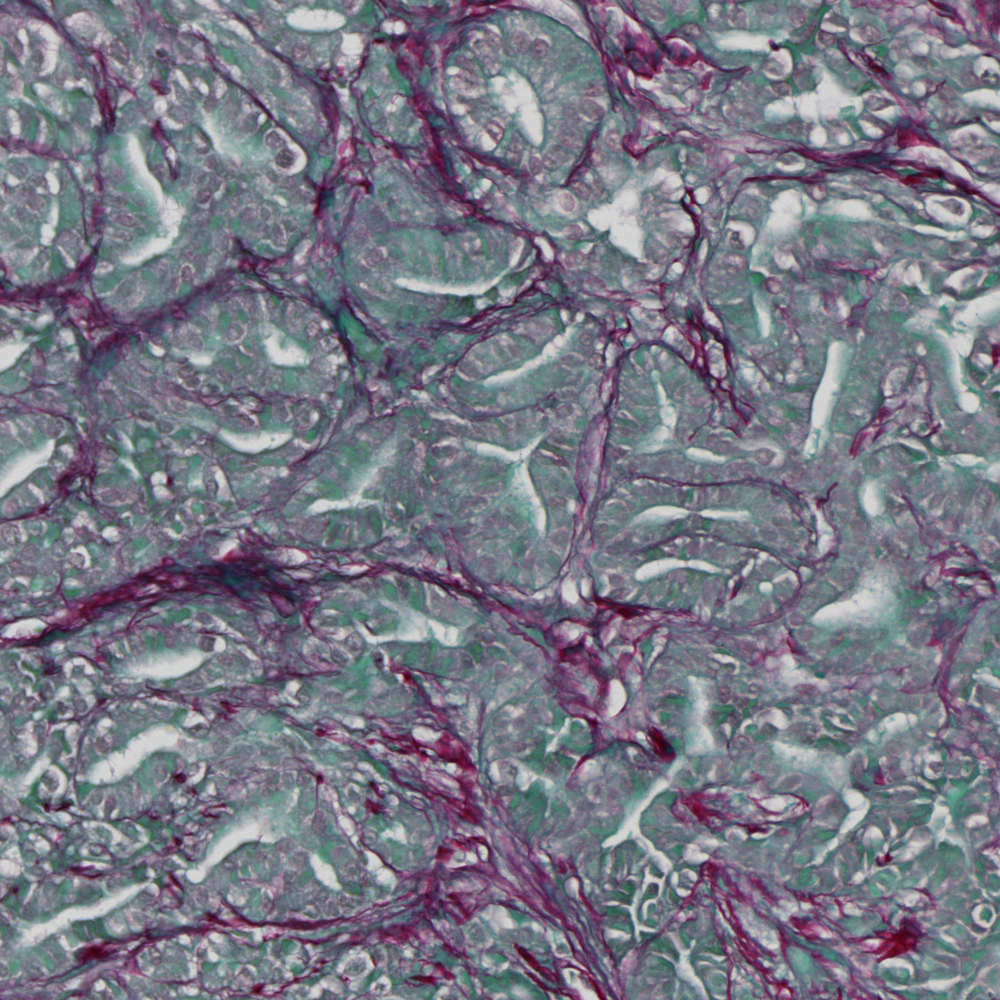

Supplement: Supplementary file 3 — Source data Fig. 1 [file 44321_2024_157_MOESM3_ESM.zip › Figure 1/1E collagen deposition/v-A12.tif]

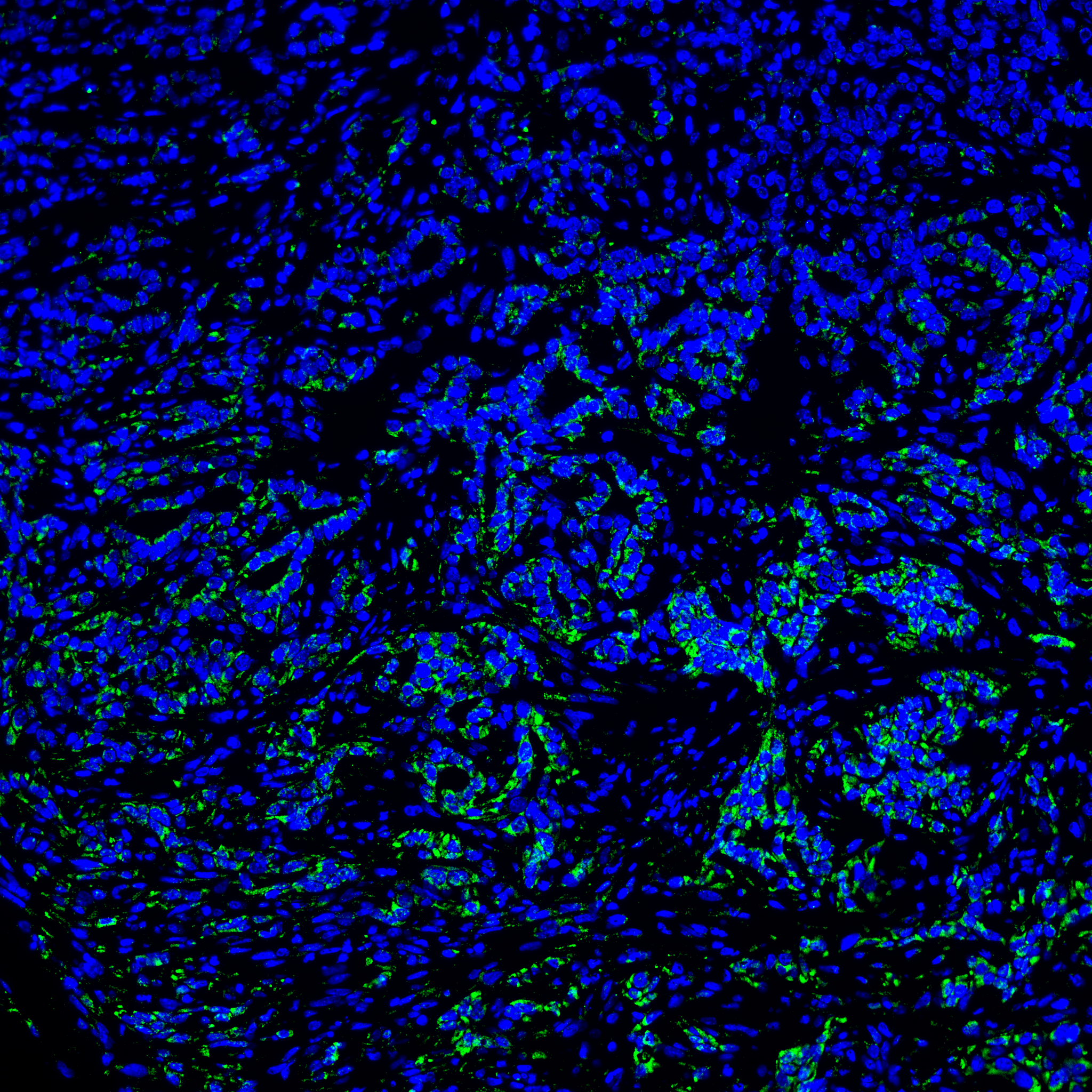

Supplement: Supplementary file 3 — Source data Fig. 1 [file 44321_2024_157_MOESM3_ESM.zip › Figure 1/1D ADAM12 quantification/v-A12_ADAM12.tif]

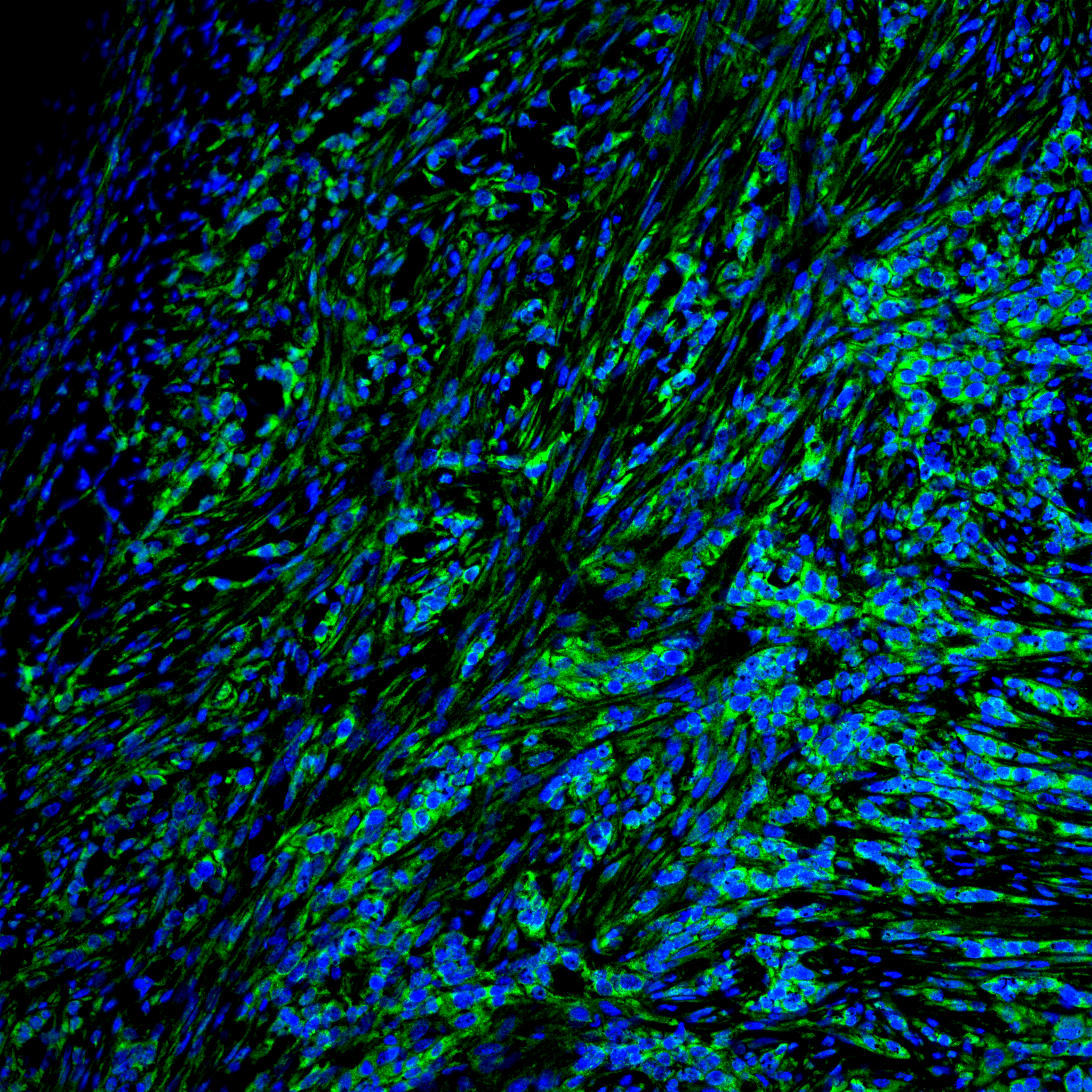

Supplement: Supplementary file 3 — Source data Fig. 1 [file 44321_2024_157_MOESM3_ESM.zip › Figure 1/1D ADAM12 quantification/v-CTRL_ADAM12 .tif]

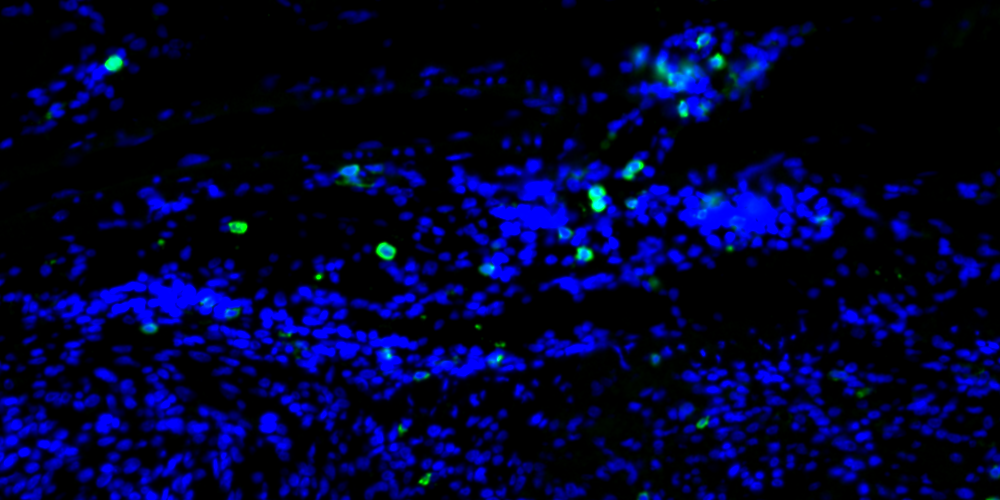

Supplement: Supplementary file 4 — Source data Fig. 2 [file 44321_2024_157_MOESM4_ESM.zip › Figure 2/2C/Edge v-CTRL.tif]

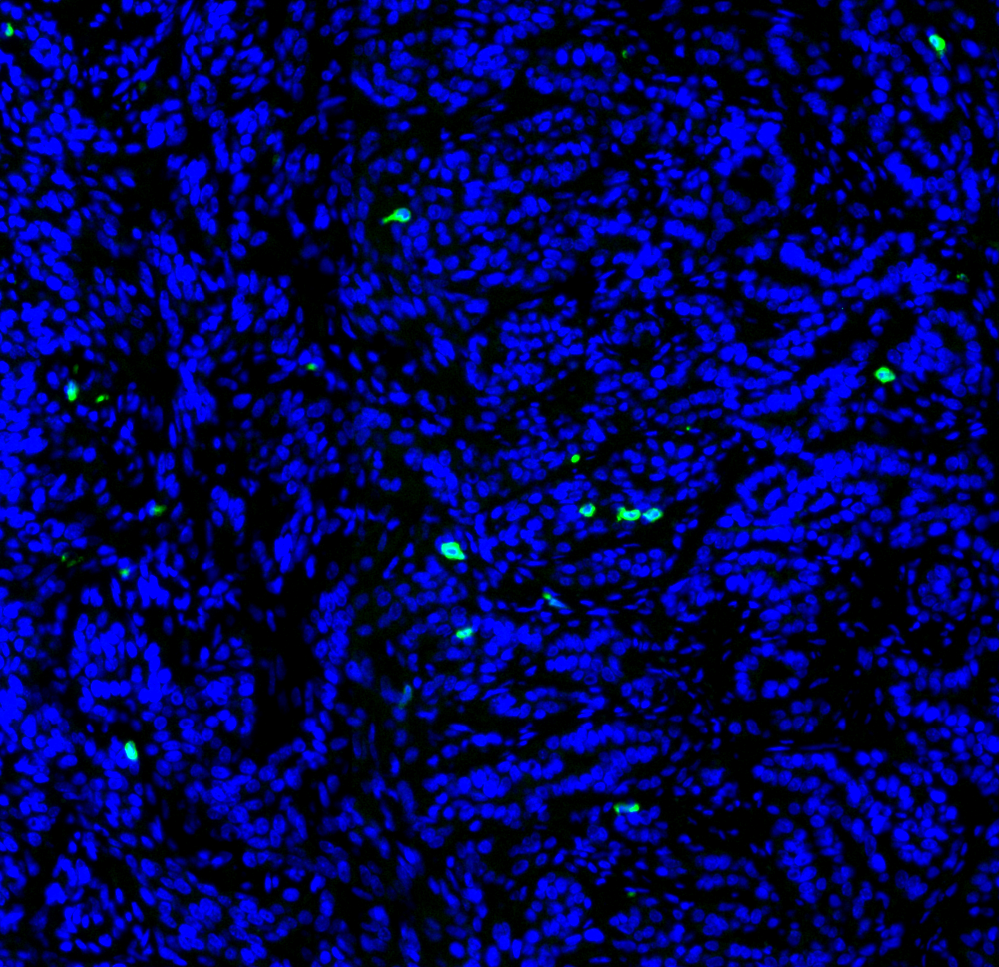

Supplement: Supplementary file 4 — Source data Fig. 2 [file 44321_2024_157_MOESM4_ESM.zip › Figure 2/2C/Interior v-CTRL.tif]

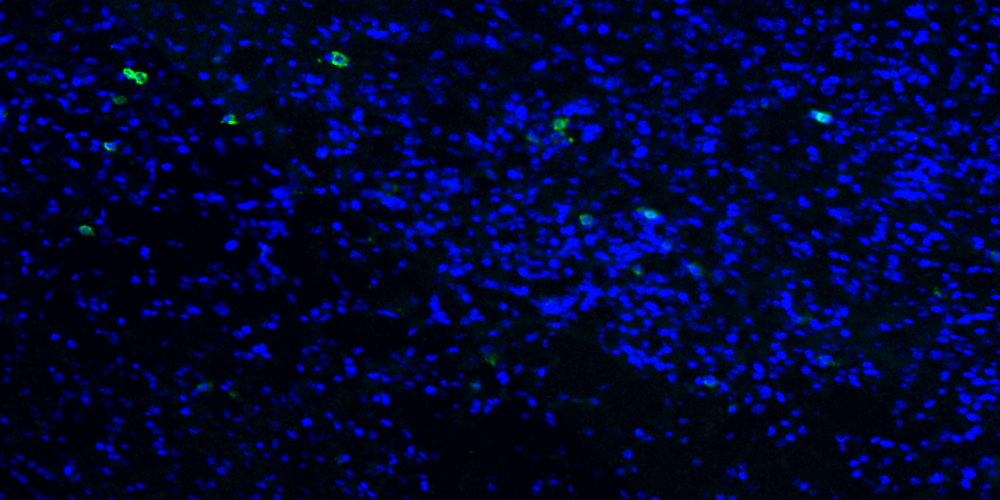

Supplement: Supplementary file 4 — Source data Fig. 2 [file 44321_2024_157_MOESM4_ESM.zip › Figure 2/2C/Edge_v-A12.tif]

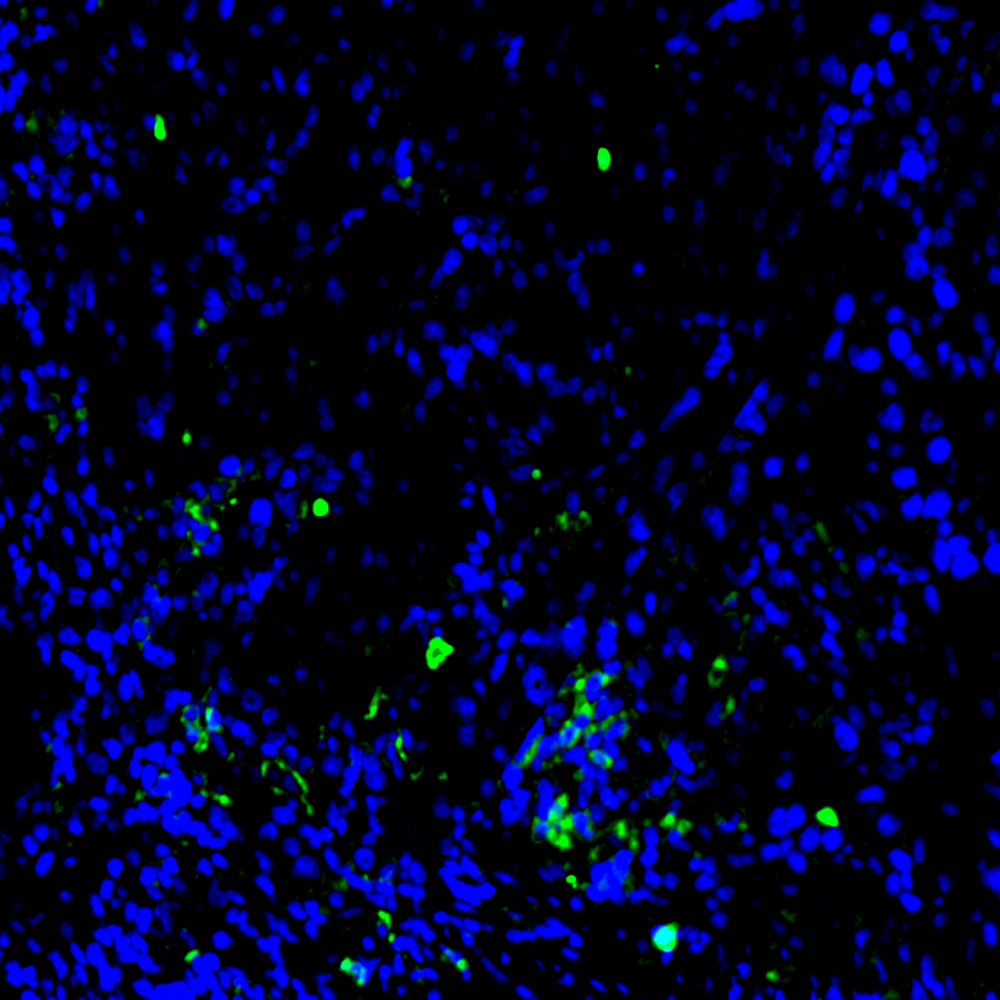

Supplement: Supplementary file 4 — Source data Fig. 2 [file 44321_2024_157_MOESM4_ESM.zip › Figure 2/2C/Interior_v-A12.tif]

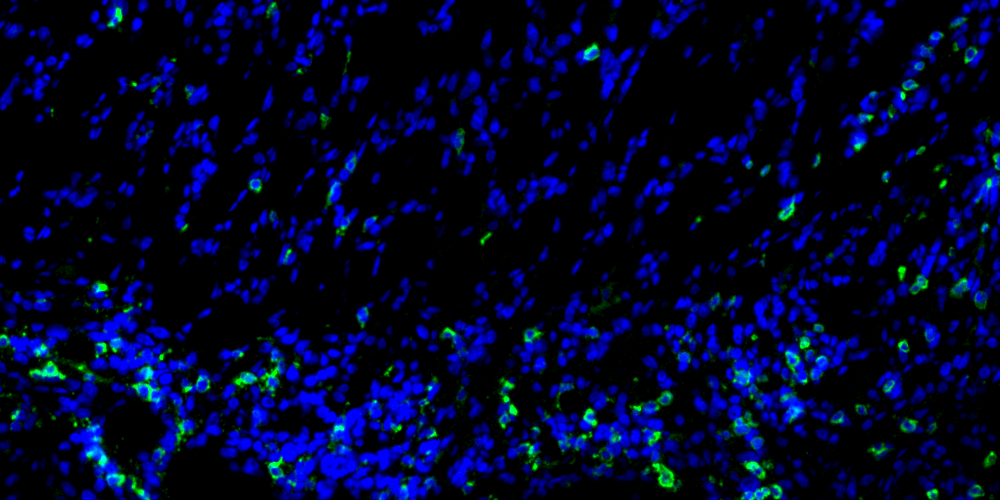

Supplement: Supplementary file 4 — Source data Fig. 2 [file 44321_2024_157_MOESM4_ESM.zip › Figure 2/2B/Edge_CD4_v-CTRL.tif]

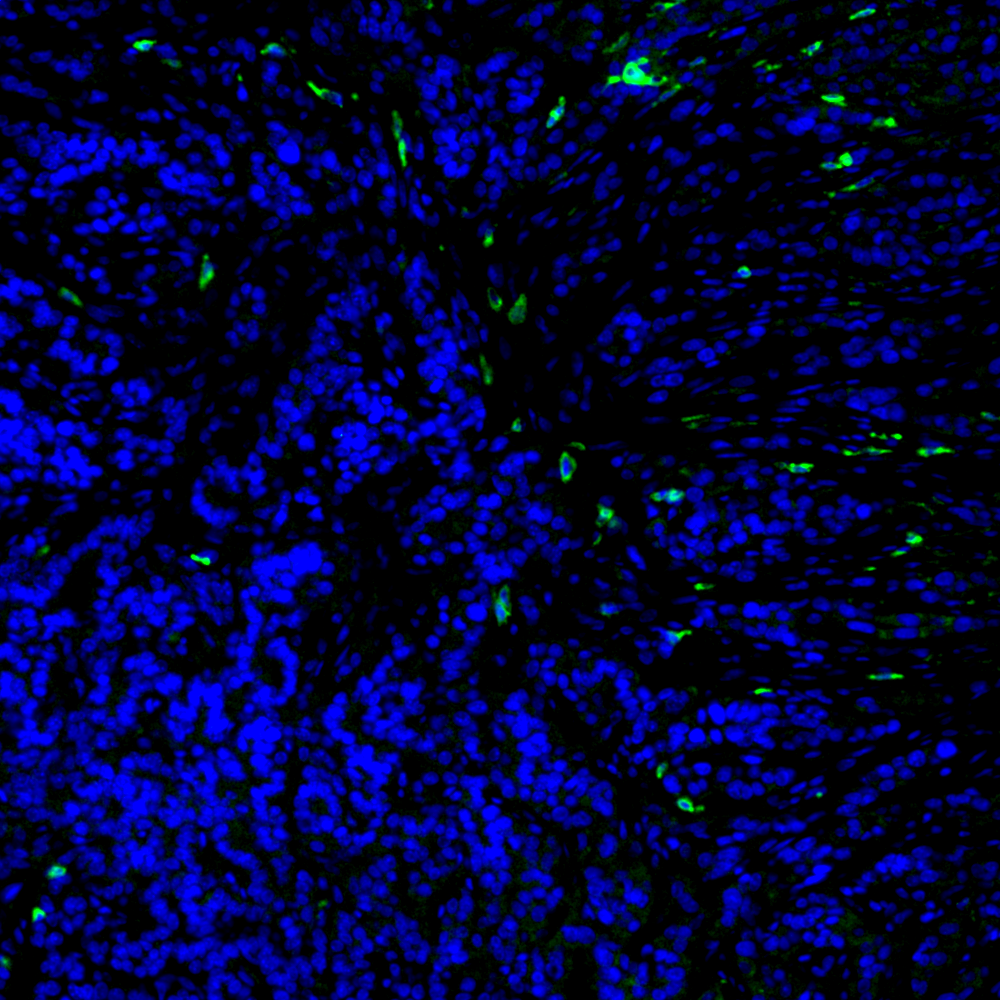

Supplement: Supplementary file 4 — Source data Fig. 2 [file 44321_2024_157_MOESM4_ESM.zip › Figure 2/2B/Interior_CD4_v-CTRL.tif]

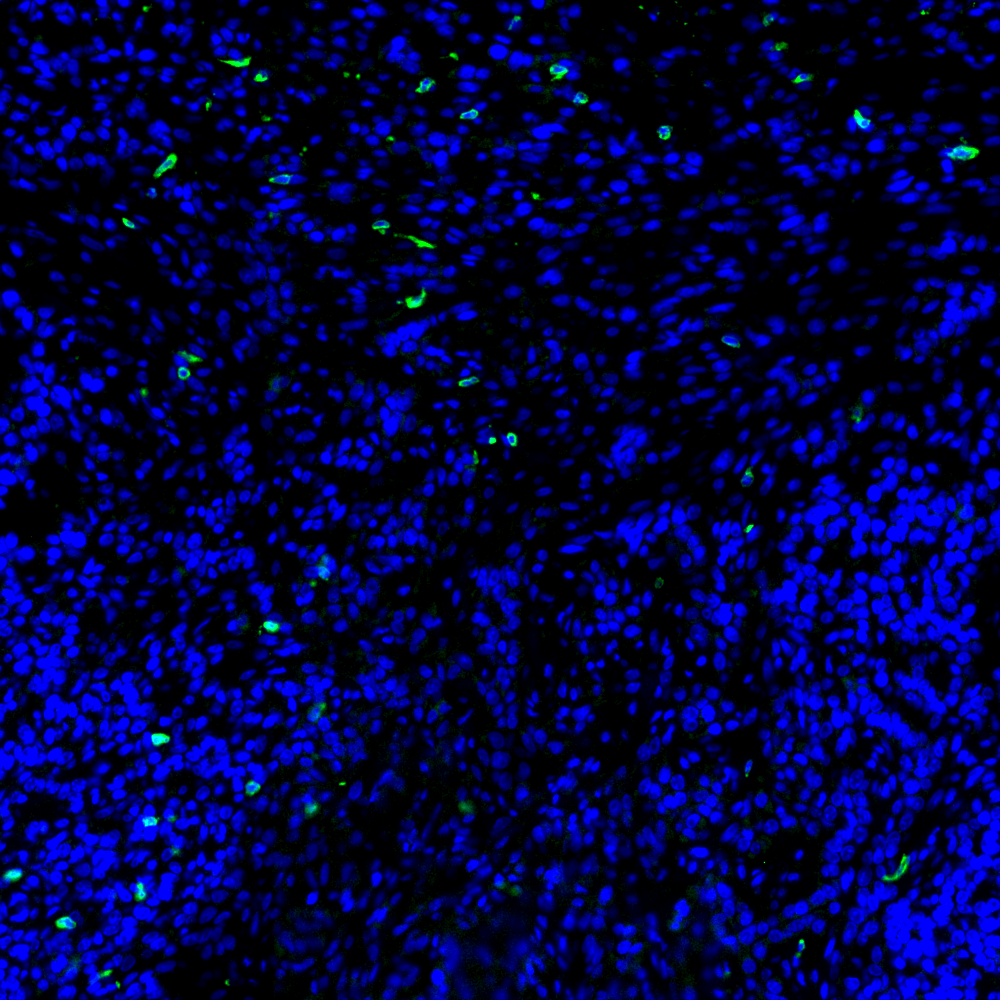

Supplement: Supplementary file 4 — Source data Fig. 2 [file 44321_2024_157_MOESM4_ESM.zip › Figure 2/2B/Interior_CD4_v-A12.tif]

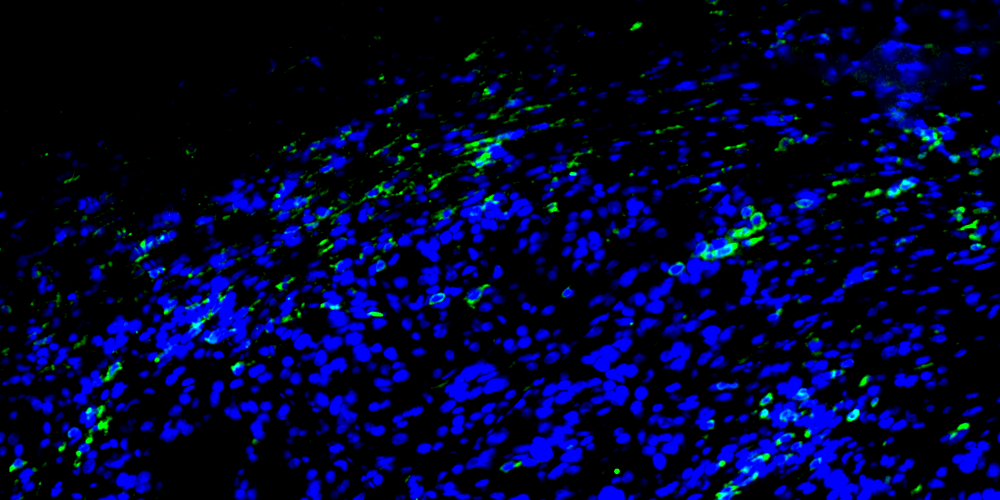

Supplement: Supplementary file 4 — Source data Fig. 2 [file 44321_2024_157_MOESM4_ESM.zip › Figure 2/2B/Edge_CD4_v-A12.tif]

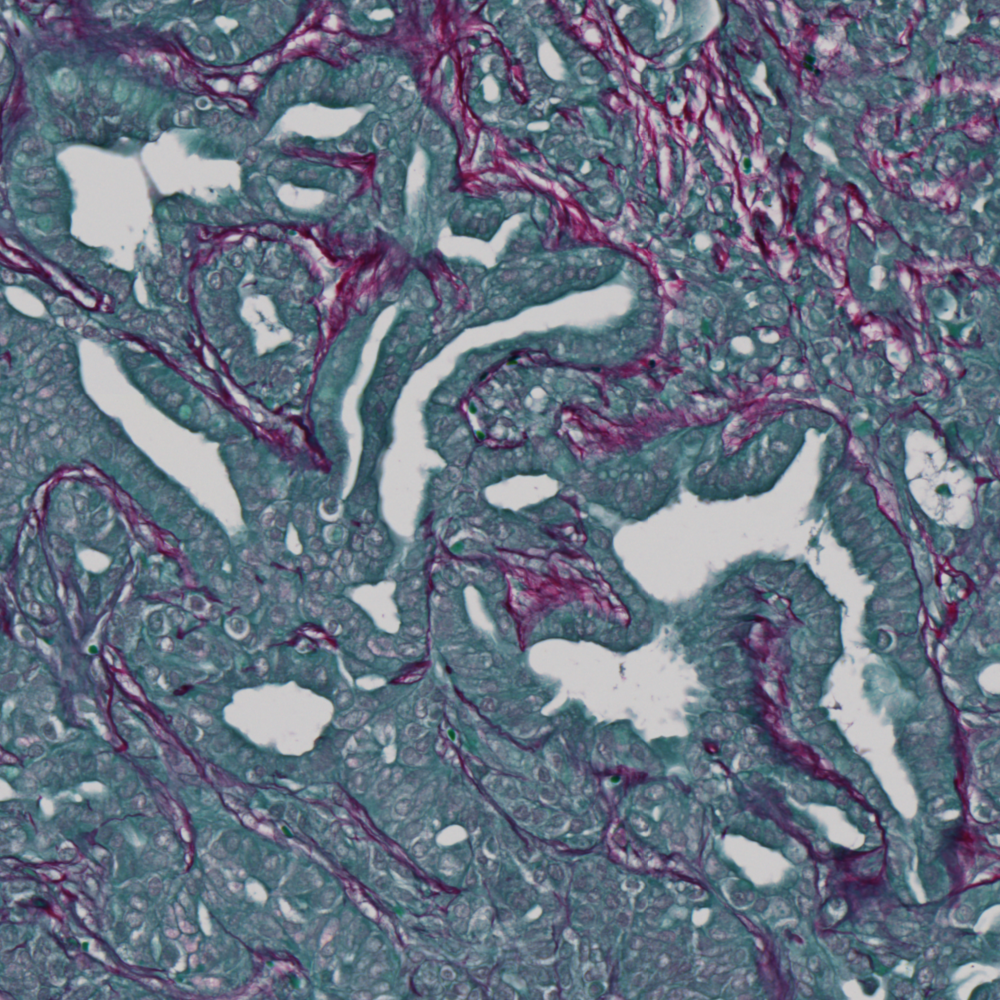

Supplement: Supplementary file 5 — Source data Fig. 3 [file 44321_2024_157_MOESM5_ESM.zip › Figure 3/3E/Th_A12_v-A12.tif]

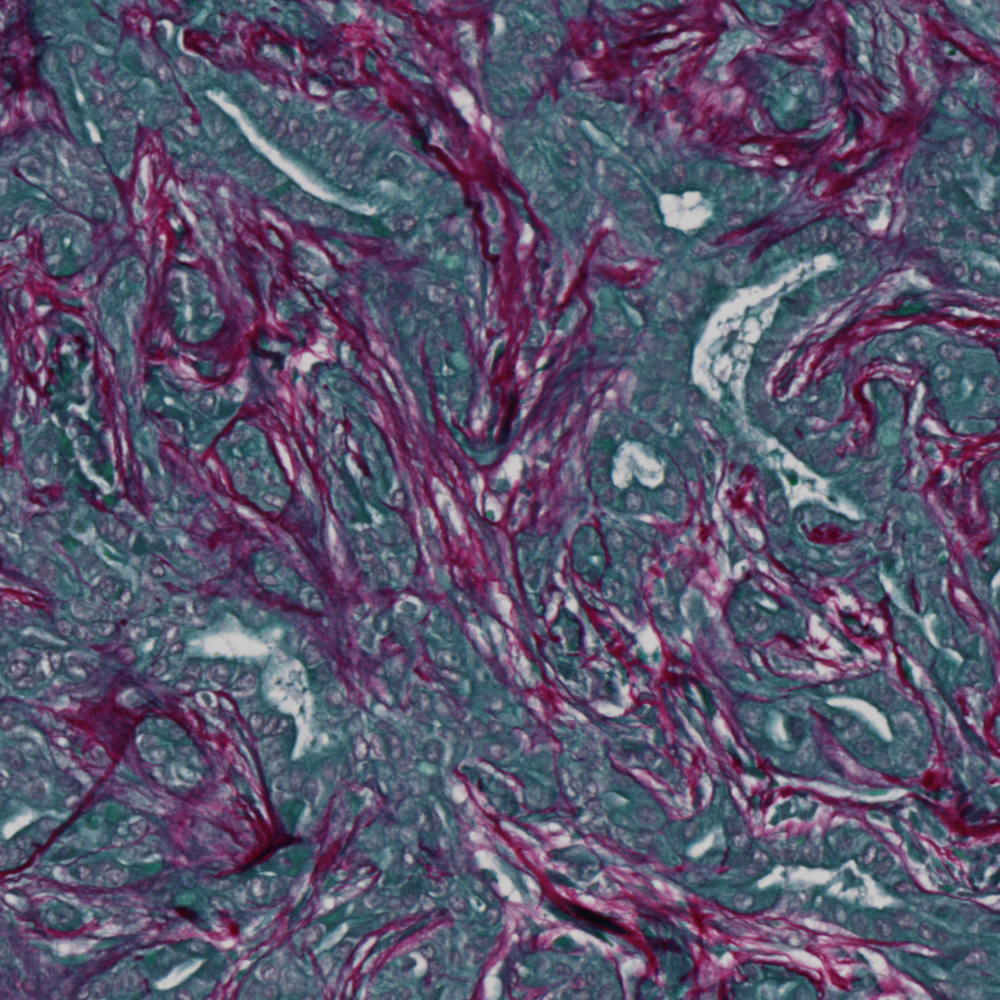

Supplement: Supplementary file 5 — Source data Fig. 3 [file 44321_2024_157_MOESM5_ESM.zip › Figure 3/3E/Th_A12_v-CTRL.tif]

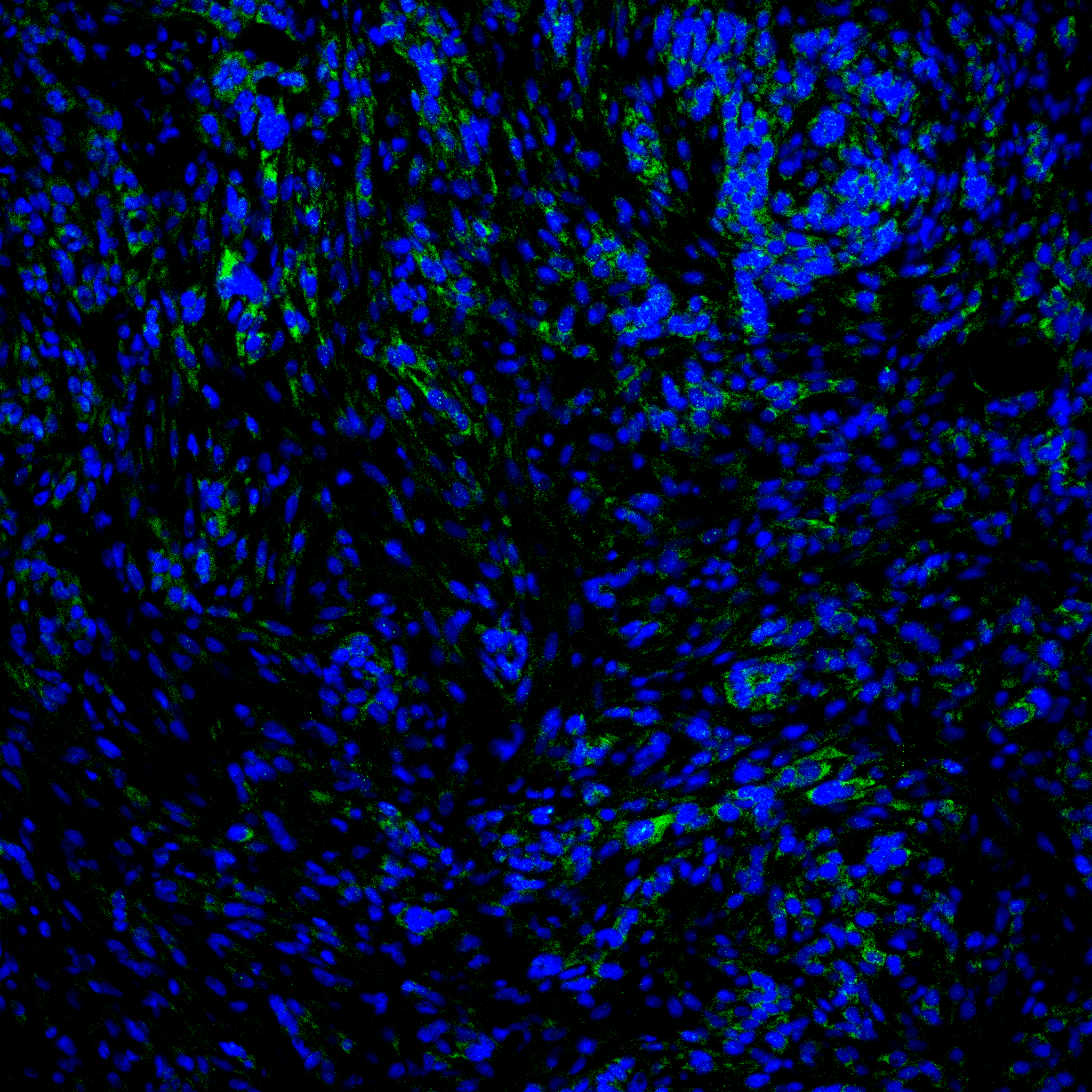

Supplement: Supplementary file 5 — Source data Fig. 3 [file 44321_2024_157_MOESM5_ESM.zip › Figure 3/3D/Th_A12_v-A12.tif]

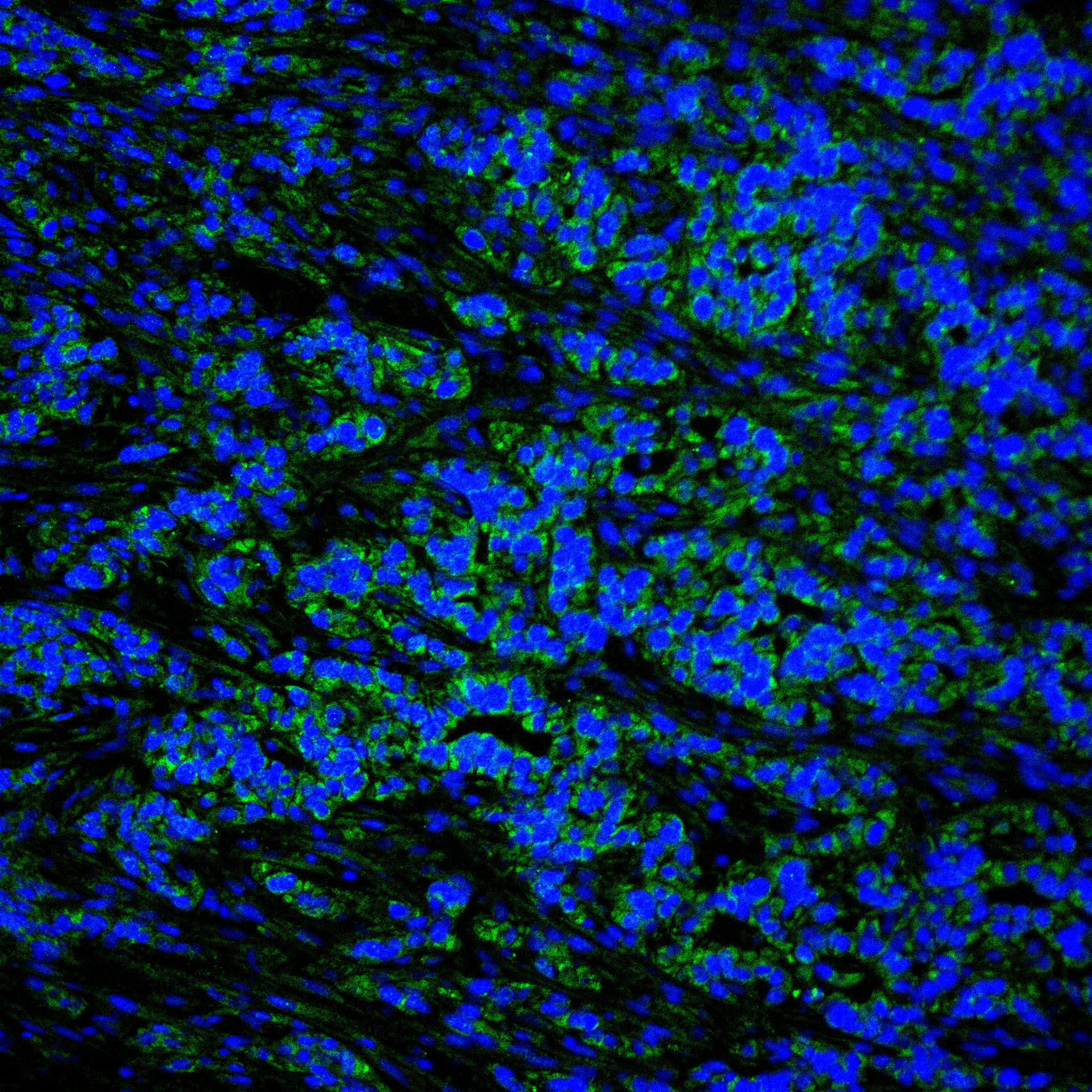

Supplement: Supplementary file 5 — Source data Fig. 3 [file 44321_2024_157_MOESM5_ESM.zip › Figure 3/3D/Th_A12_v-CTRL.tif]

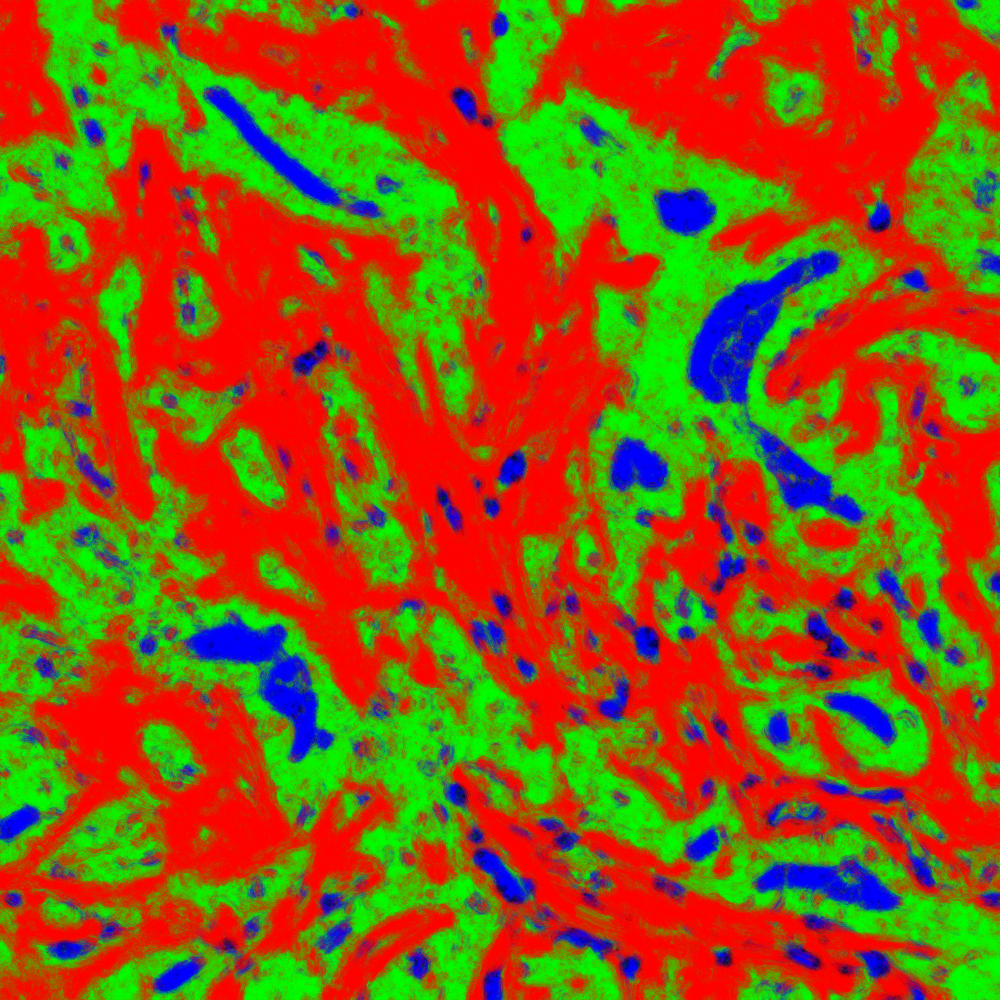

Supplement: Supplementary file 5 — Source data Fig. 3 [file 44321_2024_157_MOESM5_ESM.zip › Figure 3/3F/Th_A12_3_v-CTRL.tif]

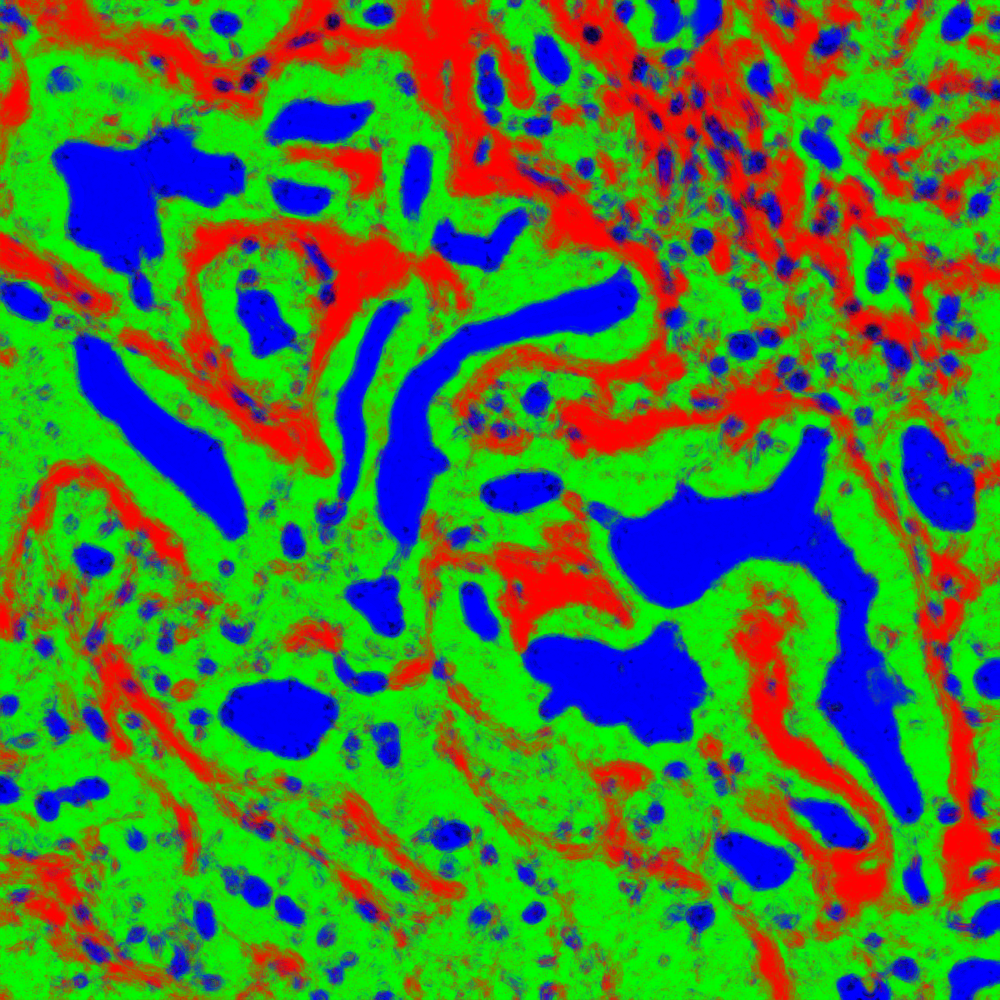

Supplement: Supplementary file 5 — Source data Fig. 3 [file 44321_2024_157_MOESM5_ESM.zip › Figure 3/3F/Th_A12_2_v-A12.tif]

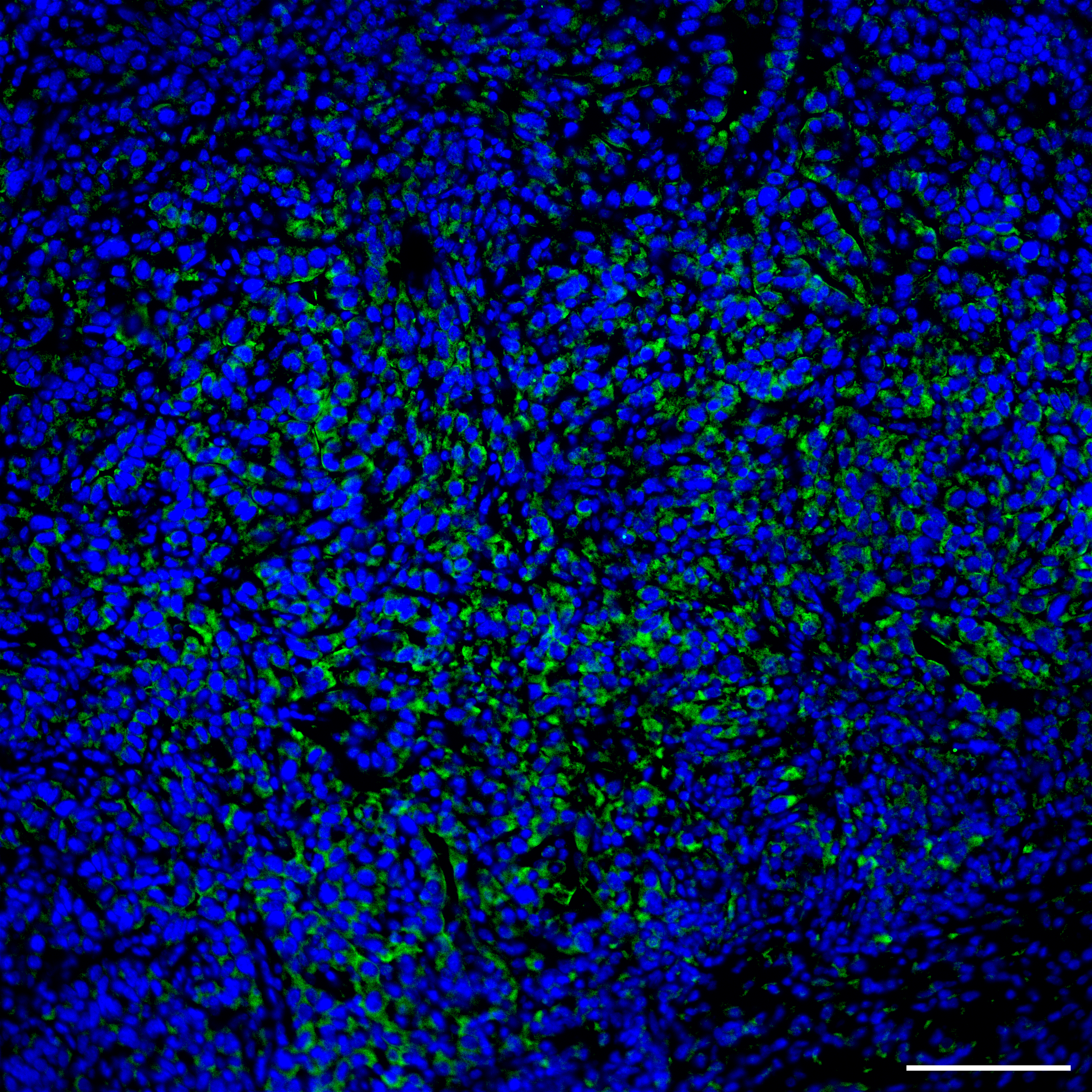

Supplement: Supplementary file 6 — Source data Fig. 4 [file 44321_2024_157_MOESM6_ESM.zip › Figure 4/4C/ADAM12 IF_v_CTRL.tif]

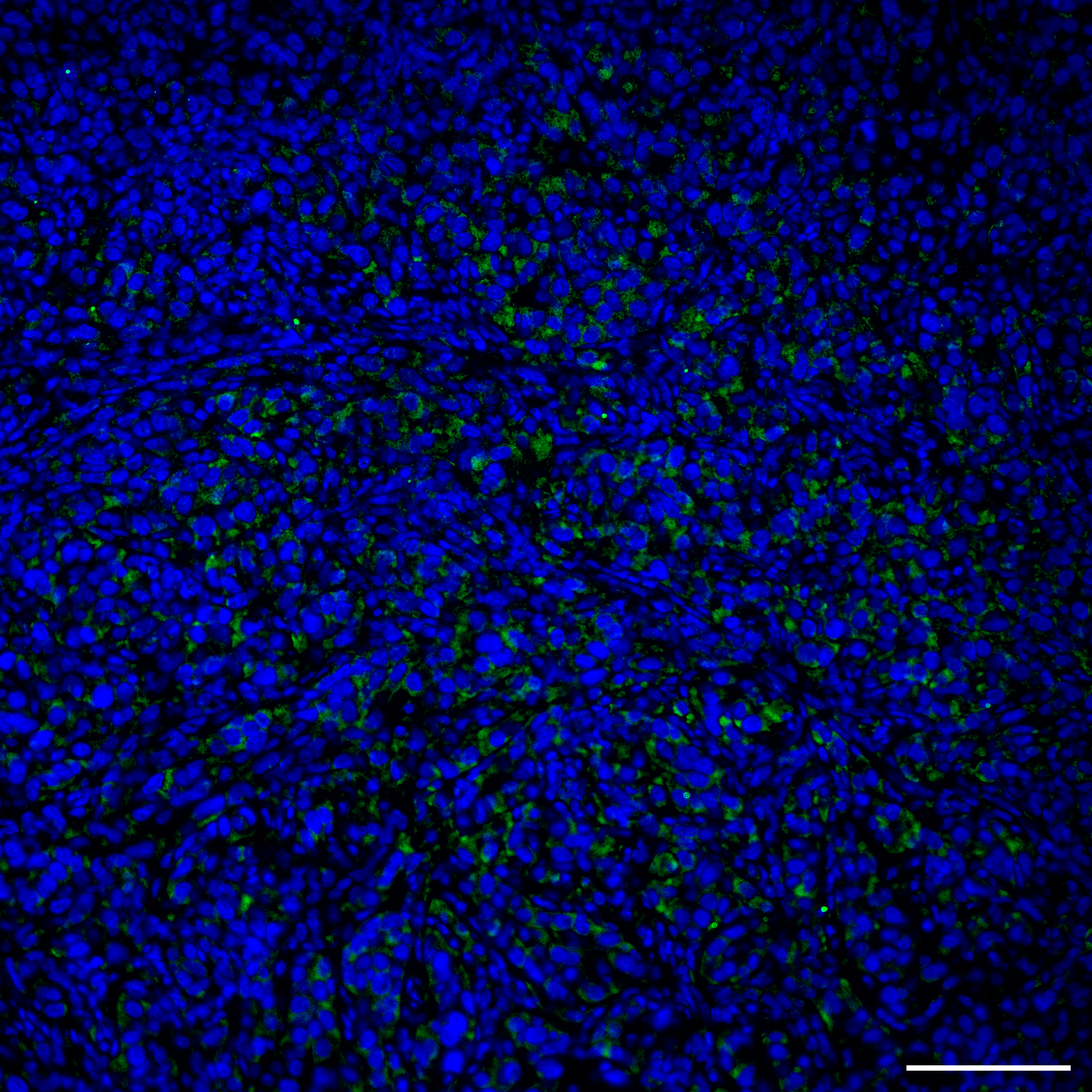

Supplement: Supplementary file 6 — Source data Fig. 4 [file 44321_2024_157_MOESM6_ESM.zip › Figure 4/4C/ADAM12 IF_v_A12.tif]

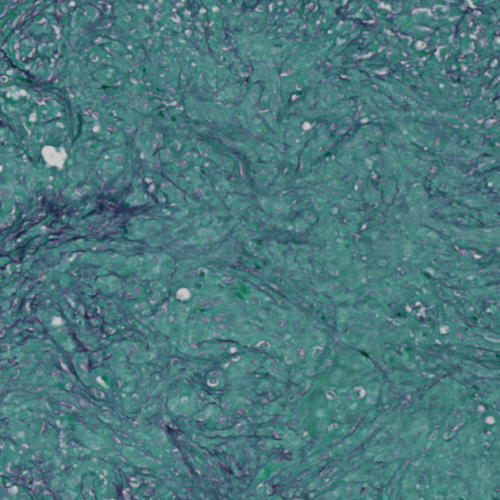

Supplement: Supplementary file 6 — Source data Fig. 4 [file 44321_2024_157_MOESM6_ESM.zip › Figure 4/4D/Collagen staining_v-A12.tif]

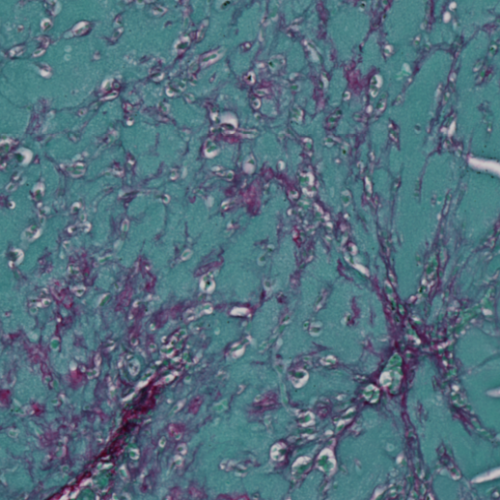

Supplement: Supplementary file 6 — Source data Fig. 4 [file 44321_2024_157_MOESM6_ESM.zip › Figure 4/4D/Collagen staining_v-CTRL.tif]

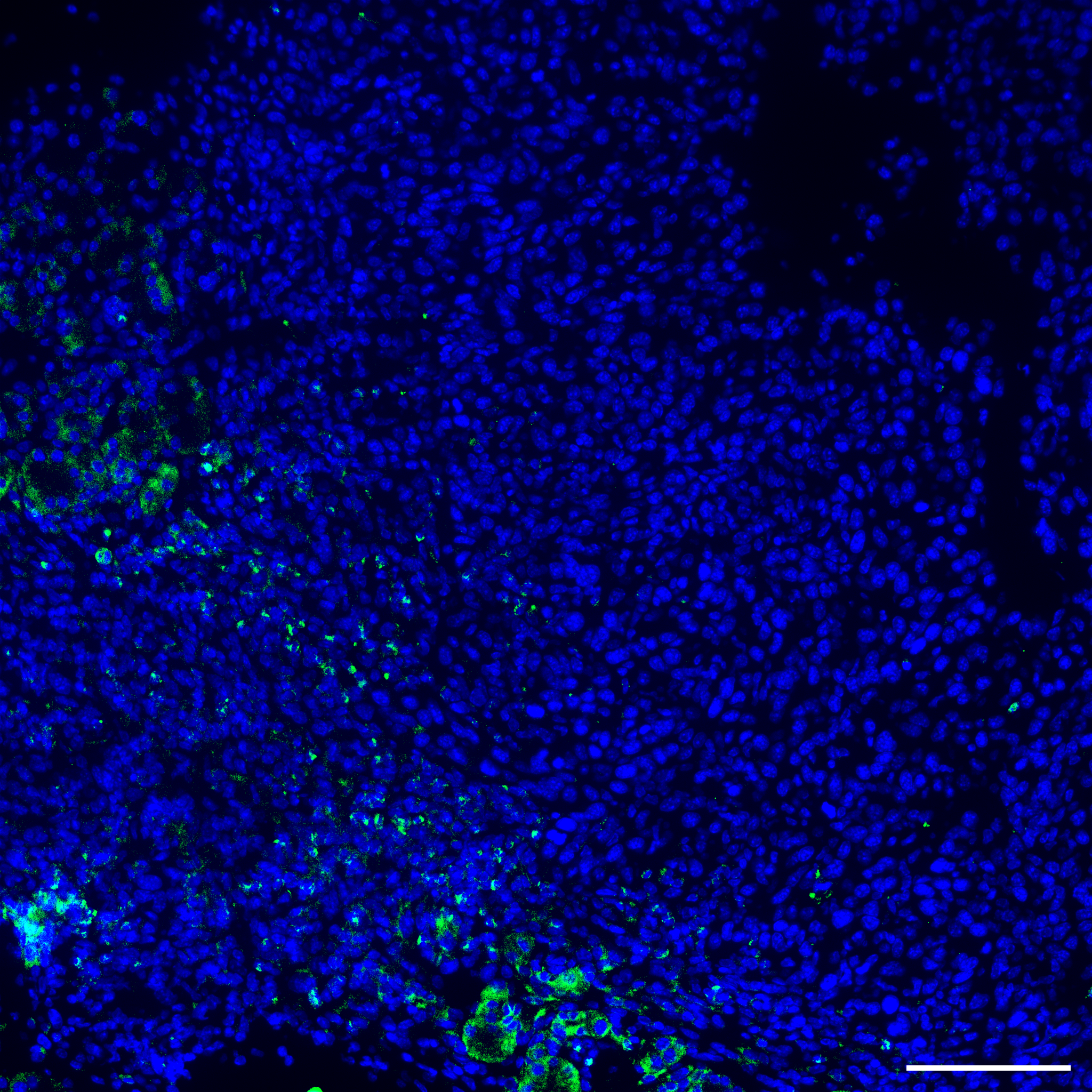

Supplement: Supplementary file 6 — Source data Fig. 4 [file 44321_2024_157_MOESM6_ESM.zip › Figure 4/4G/GLUT1 v-A12.tif]

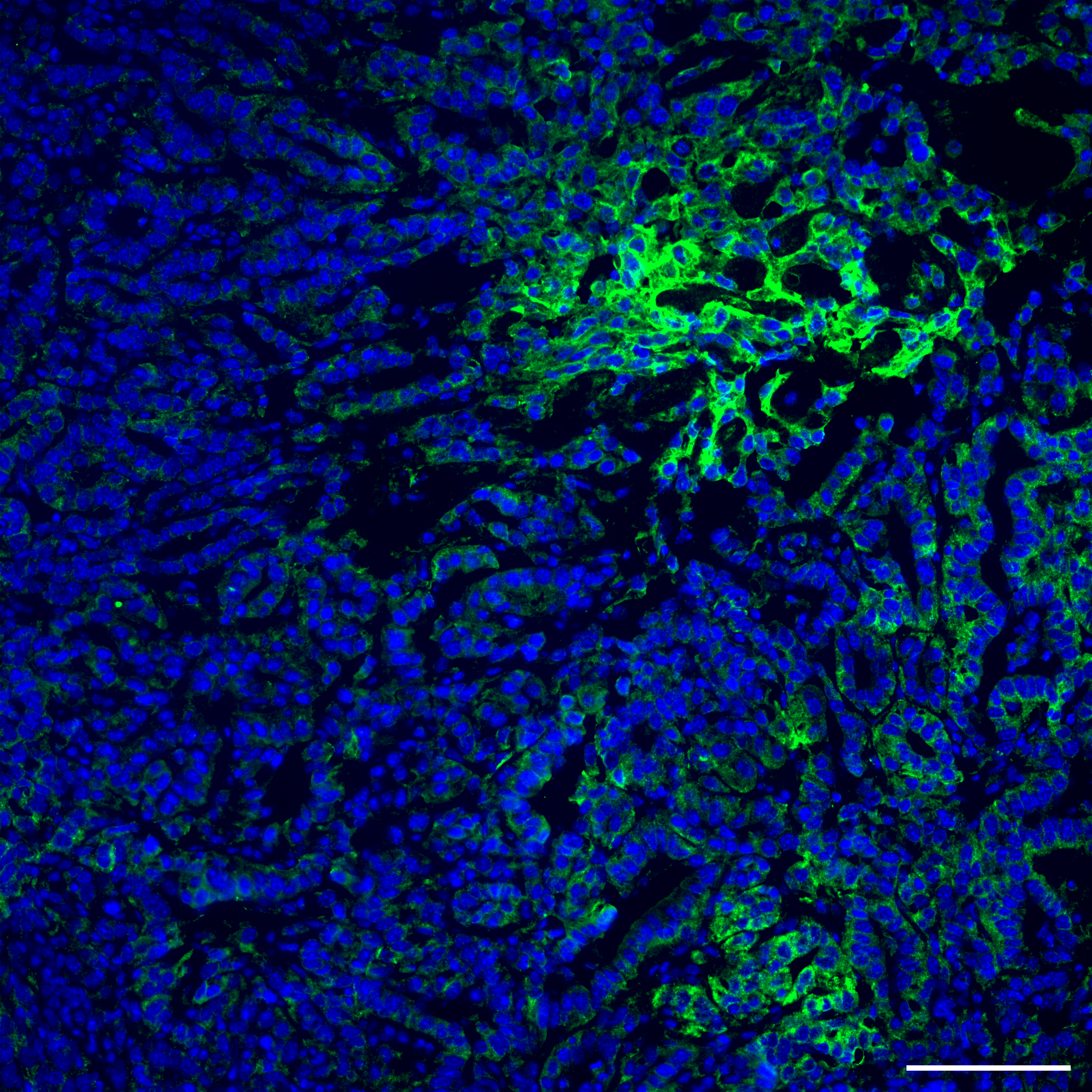

Supplement: Supplementary file 6 — Source data Fig. 4 [file 44321_2024_157_MOESM6_ESM.zip › Figure 4/4G/GLUT1 v-CTRL.tif]

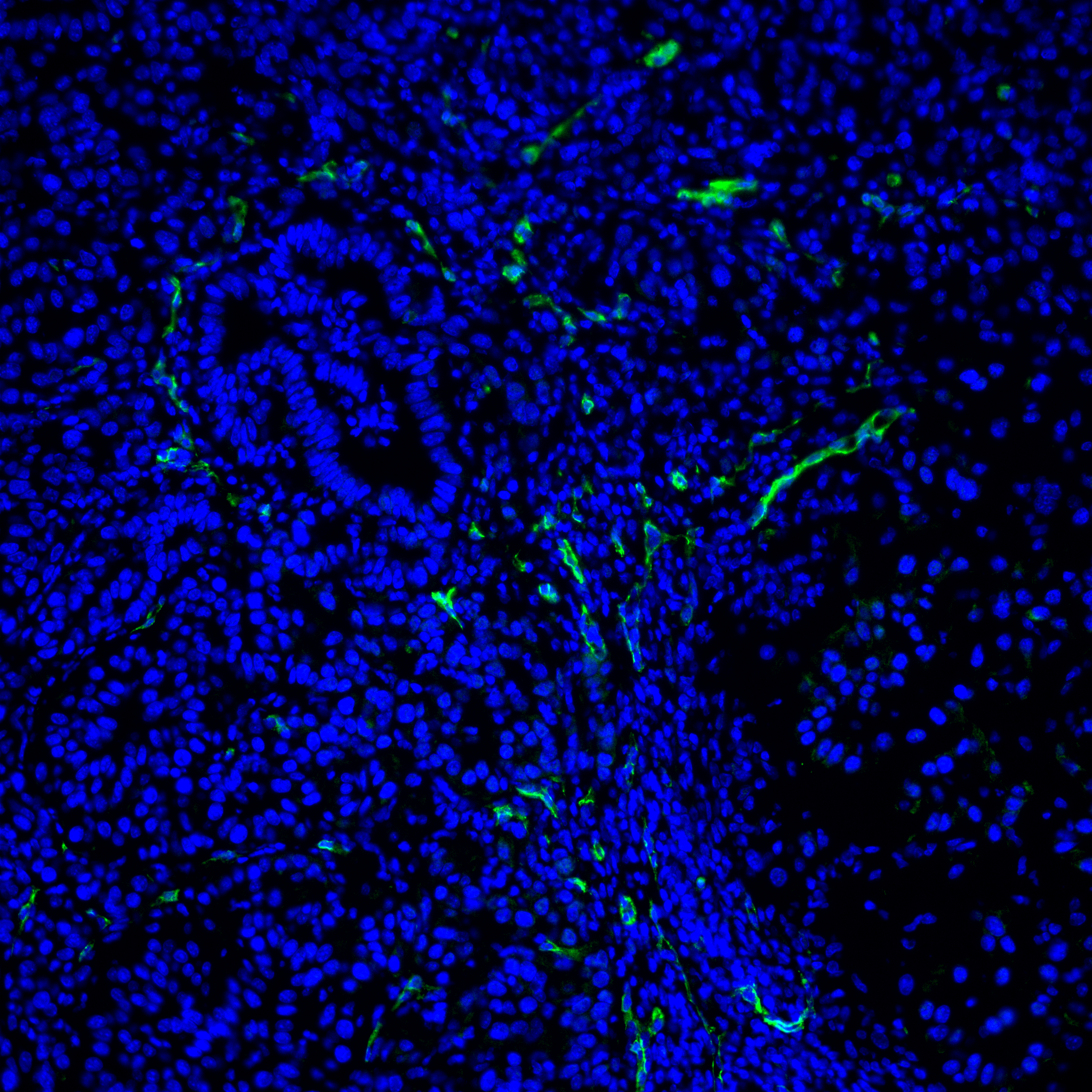

Supplement: Supplementary file 6 — Source data Fig. 4 [file 44321_2024_157_MOESM6_ESM.zip › Figure 4/4F/vascular density CD31/CD31_v-A12.tif]

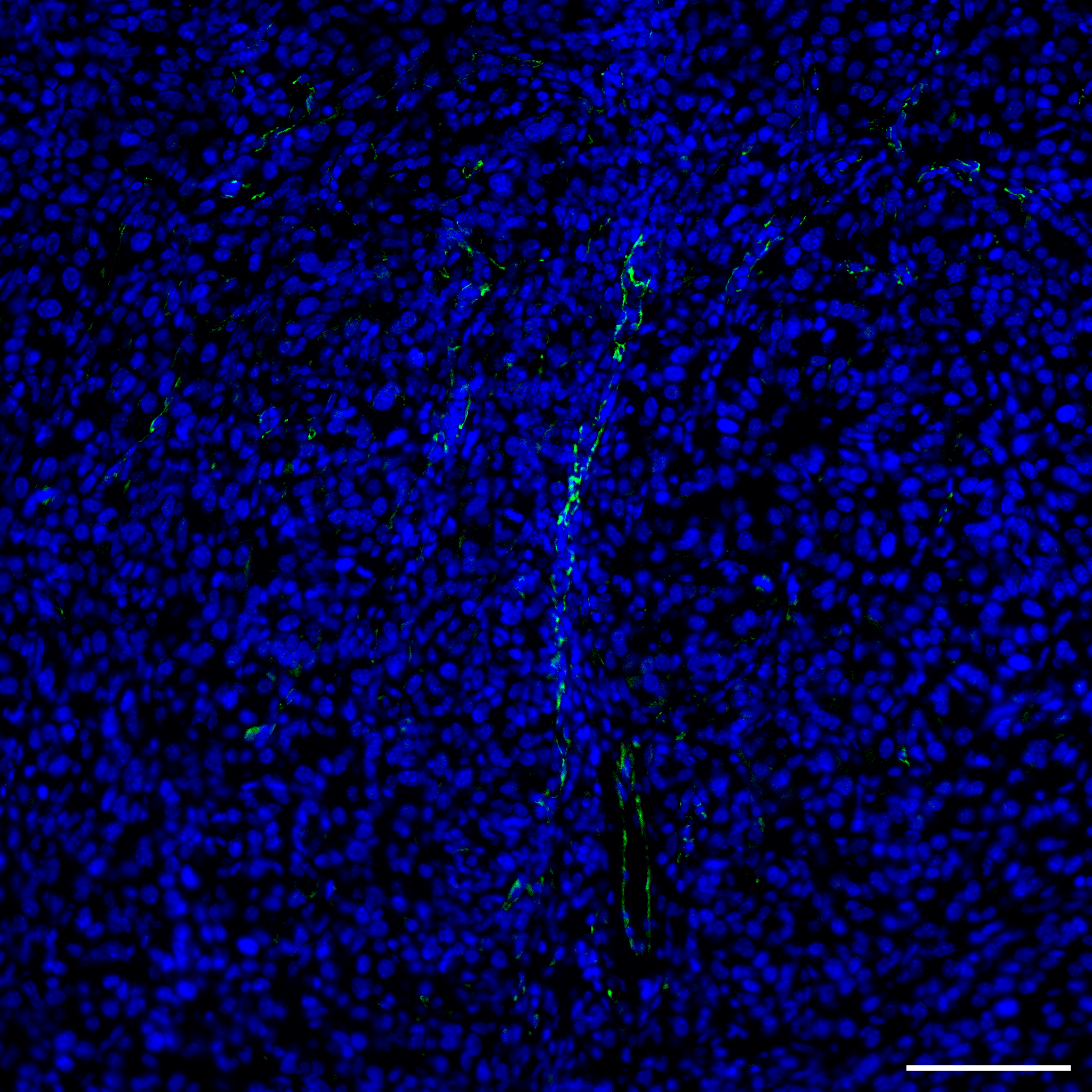

Supplement: Supplementary file 6 — Source data Fig. 4 [file 44321_2024_157_MOESM6_ESM.zip › Figure 4/4F/vascular density CD31/CD31_v-CTRL.tif]

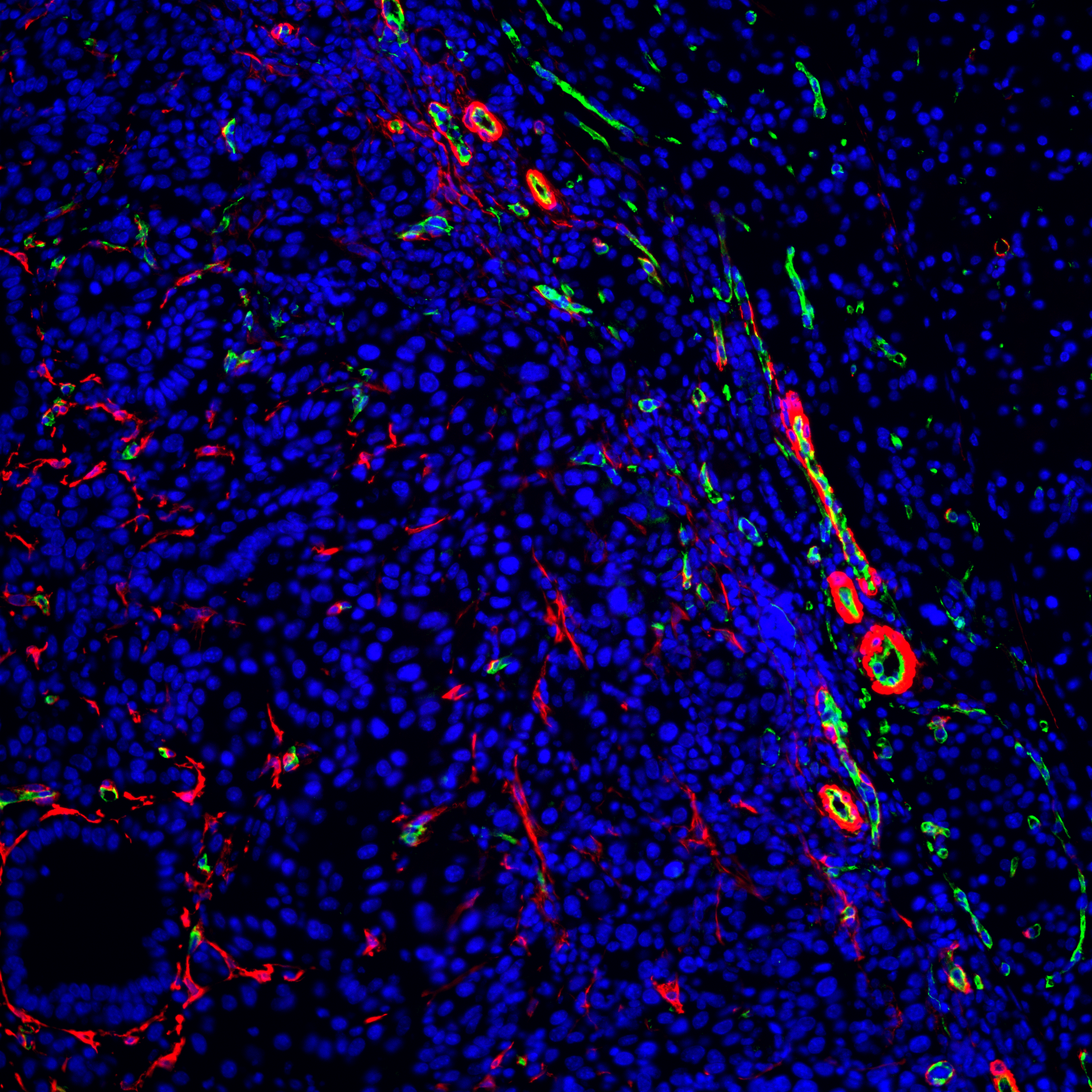

Supplement: Supplementary file 6 — Source data Fig. 4 [file 44321_2024_157_MOESM6_ESM.zip › Figure 4/4F/pericytes/Pericyte_v-A12.tif]

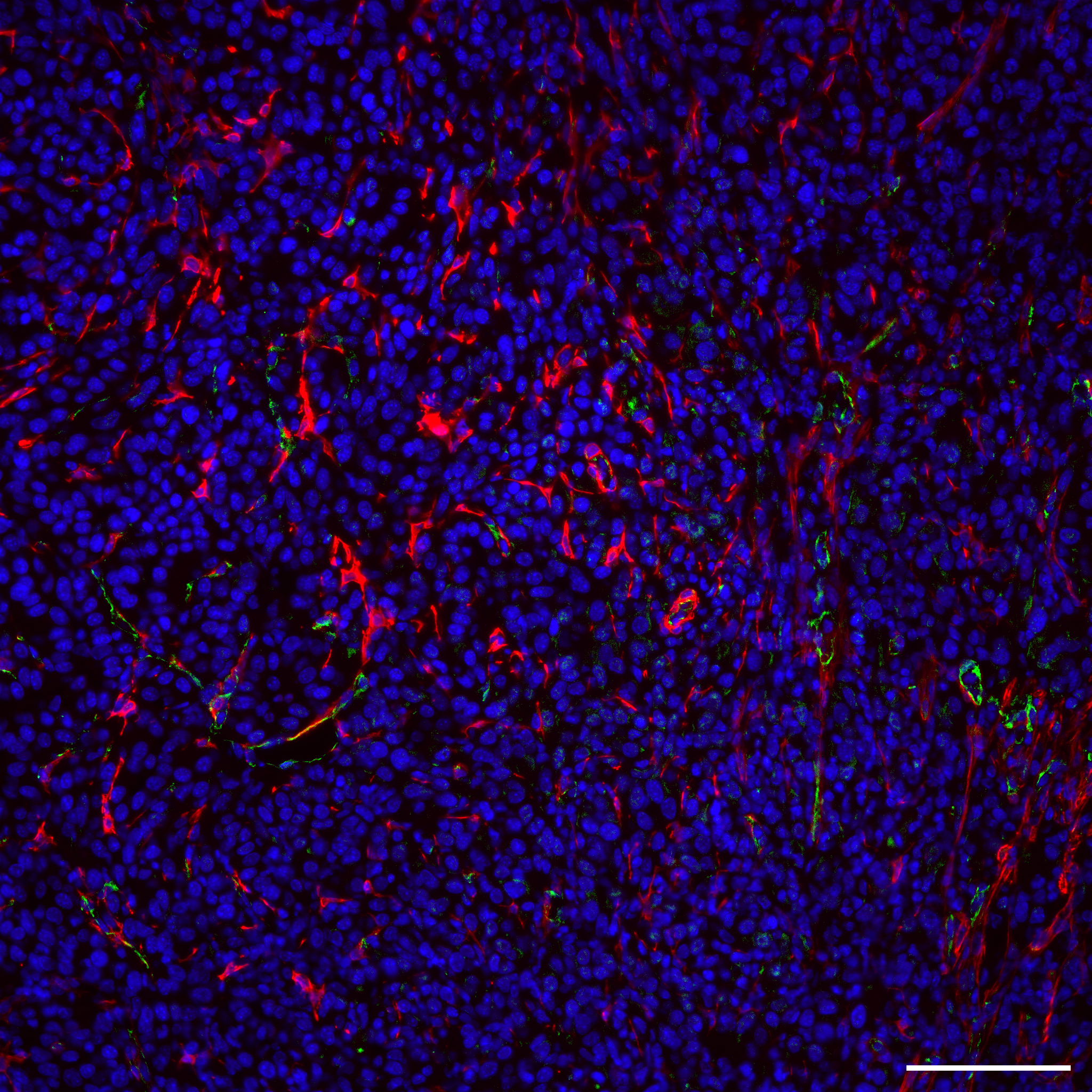

Supplement: Supplementary file 6 — Source data Fig. 4 [file 44321_2024_157_MOESM6_ESM.zip › Figure 4/4F/pericytes/Pericyte_v-CTRL.tif]
