# Supplementary material for: Fibrolytic vaccination against ADAM12 reduces desmoplasia in preclinical pancreatic adenocarcinomas
Source: EMBO Mol Med. 2024 Oct 30;16(12):3033–56. doi: 10.1038/s44321-024-00157-4 (PMC11628623; doi:10.1038/s44321-024-00157-4)
Supplement: Supplementary file 7 — Expanded View Figures [file 44321_2024_157_MOESM7_ESM.pdf]

Expanded View Figures

Figure EV1. ADAM12 expression and characterization on KP2 PDAC tumor tissue.

(**A**) Representative image of ADAM12 immunofluorescence staining (ADAM12 antibody, Invitrogen, PA5-50594) on the healthy pancreas and PDAC tumor tissue from KPPC transgenic mice. (Scale bar 100 µm). (**B**) Representative images of Sirius Red/Fast Green staining on subcutaneous KP2 PDAC sections from v-CTRL mice and v-A12 mice. (Scale bare 100 µm). (**C**) Representative image of ADAM12 immunostaining (ADAM12 antibody, Invitrogen, PA5-50594) on PDAC tumor sections from subcutaneous KP2 PDAC grafts. (Scale bar 100 µm; for the zoom-in picture, the scale bar is 50 µm). (**D**) Representative images (left) and quantitative analysis (right) of ADAM12$^+$ cell characterization, ADAM12$^+$ CAFs were indicated as ADAM12$^+$/α-SMA$^+$ (upper, left) and ADAM12$^+$/PDGFR-β$^+$ (middle, left), and ADAM12$^+$ tumor cells were indicated as ADAM12$^+$/CK19$^+$ (bottom, left) co-expression on subcutaneous KP2 PDAC tumors. ($n = 12$ fields of view from four mice. Data were presented as mean ± SEM. Scale bar is 200 µm, and the scale bar of the zoomed-in picture is 50 µm). (**E**) Vector maps of CTRL vaccine (v-CTRL) and ADAM12 vaccine (v-A12). (**F**) Quantitative analysis of ADAM12$^+$/α-SMA$^+$ (left, left), ADAM12$^+$/PDGFR-β$^+$ (left, middle), and ADAM12$^+$/CK19$^+$ (left, right) co-expression on PDAC tumors from control-vaccinated mice (v-CTRL) and ADAM12-vaccinated mice (v-A12). Data were represented as the number of cells per high power field (HPF). Representative double positive cells were indicated by white arrows respectively. ($n = 12$–16 in v-CTRL, $n = 8$–9 in v-A12. Data were presented as mean ± SEM. Statistical test: multiple *t*-test. Scale bar 50 µm).

 © The Author(s)

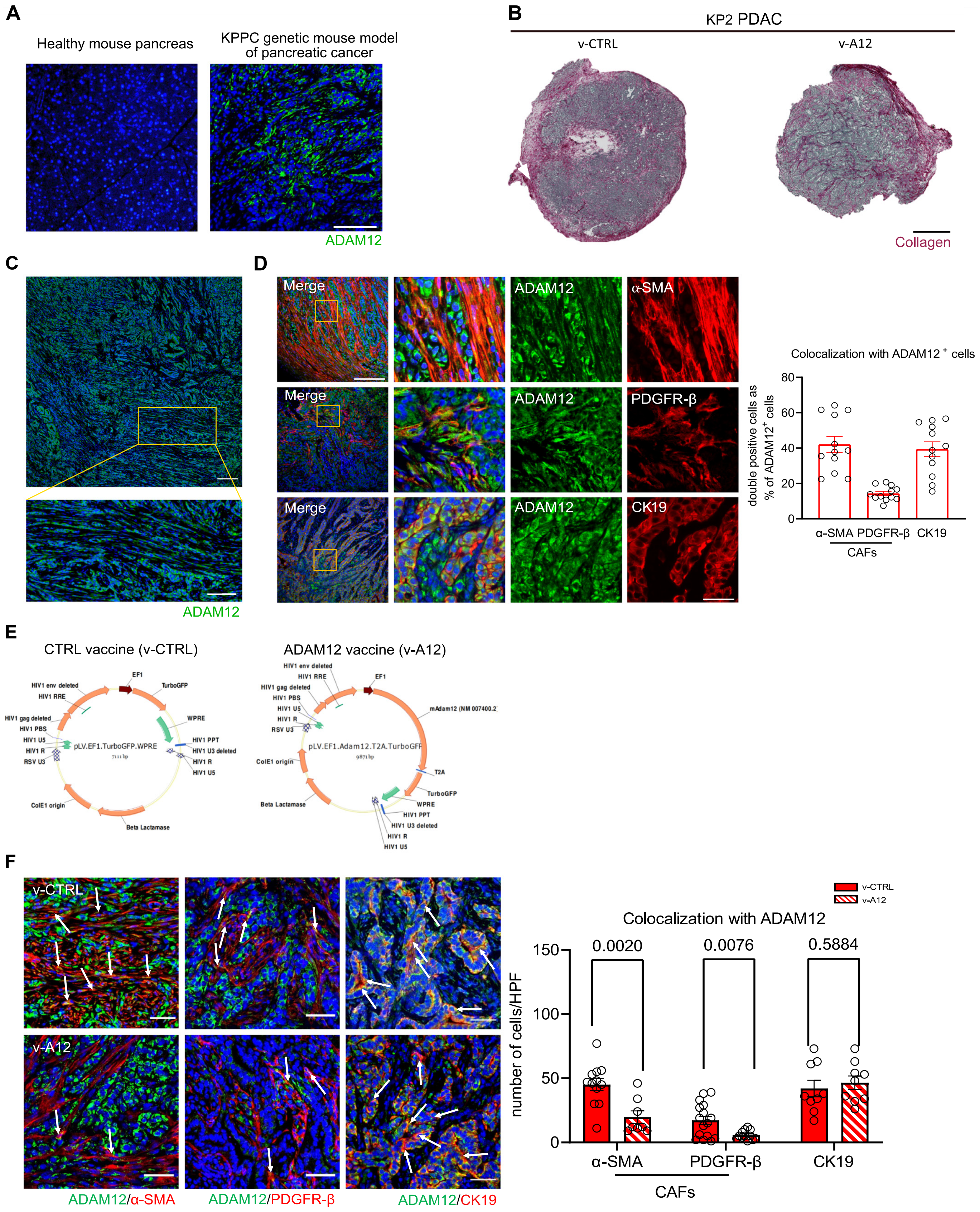
A
Healthy mouse pancreas
KPPC genetic mouse model of pancreatic cancer
ADAM12
B
KP2 PDAC
v-CTRL
v-A12
Collagen
C
ADAM12
D
Merge
ADAM12
α-SMA
PDGFR-β
CK19
Colocalization with ADAM12 + cells
double positive cells as % of ADAM12+ cells
0
20
40
60
80
α-SMA PDGFR-β CK19
CAFs
E
CTRL vaccine (v-CTRL)
ADAM12 vaccine (v-A12)
pLV.EF1.TurboGFP.WPRE
pLV.EF1.Adam12.T2A.TurboGFP
EF1
TurboGFP
WPRE
T2A
ColE1 origin
Beta Lactamase
F
v-CTRL
v-A12
ADAM12/α-SMA
ADAM12/PDGFR-β
ADAM12/CK19
v-CTRL
v-A12
Colocalization with ADAM12
number of cells/HPF
0
50
100
150
0.0020
0.0076
0.5884
α-SMA
PDGFR-β
CK19
CAFs

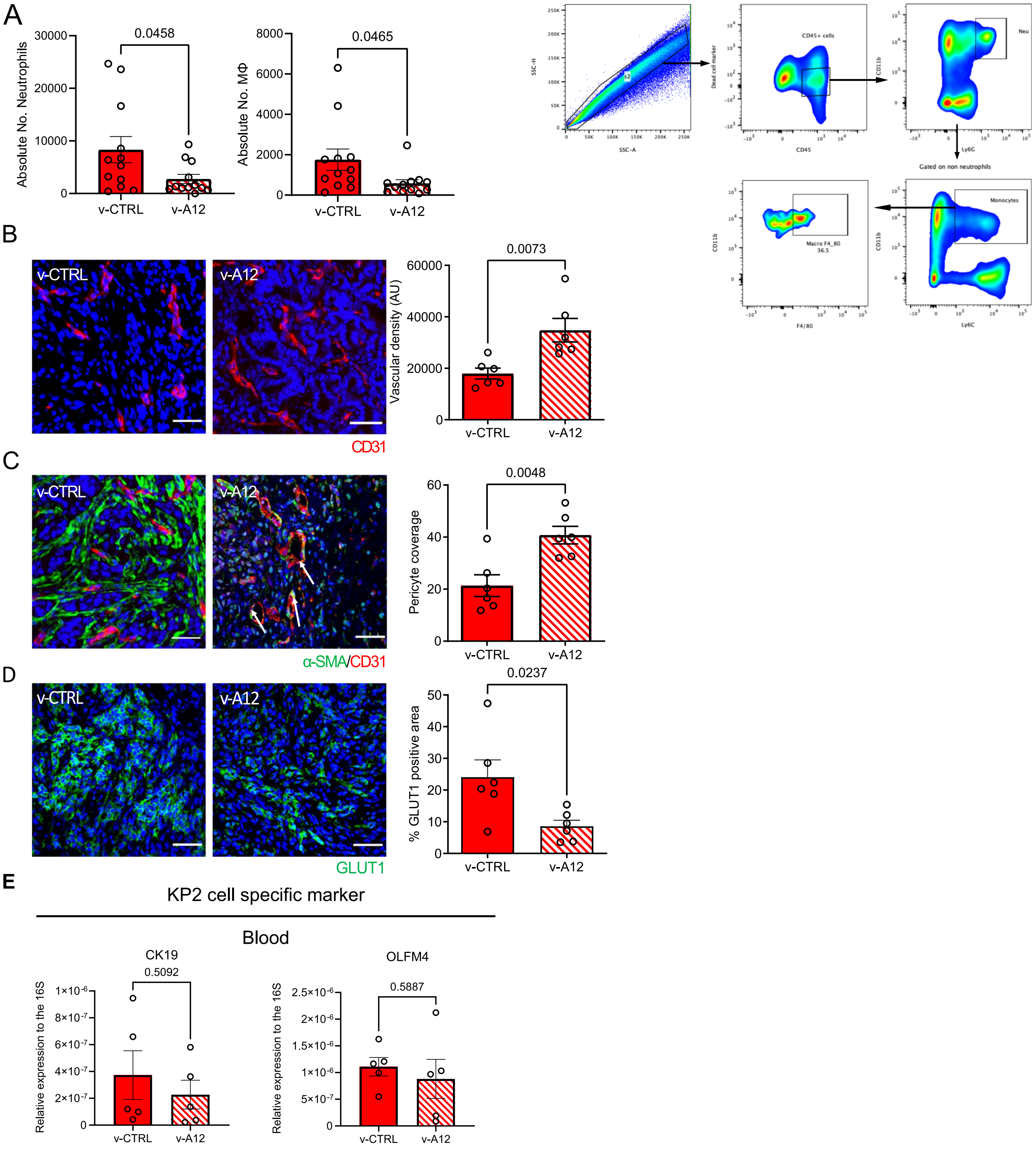
A
Absolute No. Neutrophils
30000
20000
10000
0
0.0458
v-CTRL
v-A12
Absolute No. MΦ
8000
6000
4000
2000
0
0.0465
v-CTRL
v-A12
Gated on non neutrophils
B
v-CTRL
v-A12
CD31
Vascular density (AU)
60000
40000
20000
0
0.0073
v-CTRL
v-A12
C
v-CTRL
v-A12
α-SMA/CD31
Pericyte coverage
60
40
20
0
0.0048
v-CTRL
v-A12
D
v-CTRL
v-A12
GLUT1
% GLUT1 positive area
50
40
30
20
10
0
0.0237
v-CTRL
v-A12
E
KP2 cell specific marker
Blood
CK19
0.5092
Relative expression to the 16S
1×10⁻⁶
8×10⁻⁷
6×10⁻⁷
4×10⁻⁷
2×10⁻⁷
0
v-CTRL
v-A12
OLFM4
0.5887
Relative expression to the 16S
2.5×10⁻⁶
2×10⁻⁶
1.5×10⁻⁶
1×10⁻⁶
5×10⁻⁷
0
v-CTRL
v-A12

© The Author(s)

Figure EV2. Prophylactic ADAM12 vaccination increased tumor vascular density and pericyte coverage.

(**A**) Quantitative analysis of the absolute number of neutrophils (left) and macrophages (middle) within PDAC tumor tissues from control-vaccinated mice (v-CTRL) and ADAM12-vaccinated mice (v-A12); corresponding flow cytometry gating strategy of myeloid cells (right): neutrophils were identified as $CD45^+CD11b^+Ly6G^+$ cells; monocytes were gated based on non-neutrophils (negative gating), and then identified as $CD11b^+Ly6C^+$ population. Then, monocyte macrophages were further defined with an $F4/80^+$ signal. ($n = 12$ mice in v-CTRL, $n = 12$ mice in v-A12. Data were presented as mean ± SEM. Statistical test: unpaired two-tailed Student's *t*-test). (**B**) Representative immunofluorescence staining images (left) and quantitative analysis of CD31-positive endothelial cells (right) on PDAC tumors from mice treated with either control vaccine (v-CTRL) or ADAM12 vaccine (v-A12). ($n = 6$ mice in v-CTRL, $n = 6$ mice in v-A12. Data were presented as mean ± SEM. Statistical test: unpaired two-tailed Student's *t*-test. Scale bar 50 µm). (**C**) Representative immunofluorescence staining images (left) and quantitative analysis of pericyte coverage assessed by α-SMA/CD31 co-localization on PDAC tumors with either control vaccine (v-CTRL) or ADAM12 vaccine (v-A12) and representative images of α-SMA/CD31 immunostaining. ($n = 6$ mice in v-CTRL, $n = 6$ mice in v-A12. Data were presented as mean ± SEM. Statistical test: unpaired two-tailed Student's *t*-test. Scale bar 50 µm). (**D**) Representative immunofluorescence staining images (left) and quantitative analysis of tumor hypoxia via GLUT1-positive cells on subcutaneous PDAC from v-CTRL mice and v-A12 mice together with representative images of GLUT1 immunostaining. ($n = 6$ mice in v-CTRL, $n = 6$ mice in v-A12. Data were presented as mean ± SEM. Statistical test: unpaired two-tailed Student's *t*-test. Scale bar 50 µm). (**E**) Gene expression analysis of the KP2-specific gene CK19 in peripheral blood at endpoint (day 28 post tumor inoculation) from subcutaneous PDAC mouse model with control vaccine (v-CTRL) or ADAM12 vaccine (v-A12). ($n = 5$ mice in v-CTRL, $n = 5$ mice in v-A12. Data were presented as mean ± SEM. Statistical test: unpaired two-tailed Student's *t*-test).

© The Author(s)

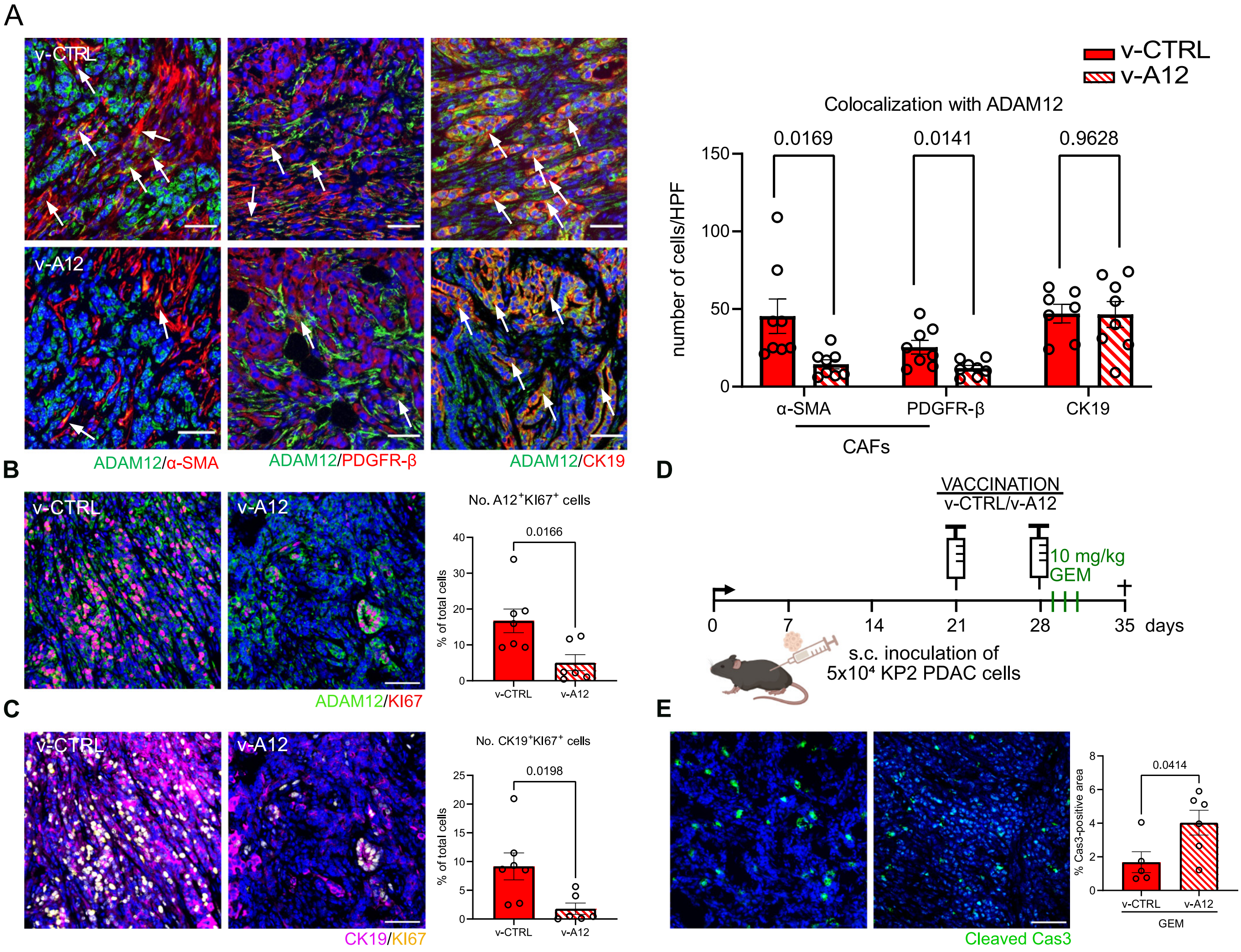

Figure EV3. Therapeutic ADAM12 vaccination targets ADAM12⁺ CAFs and inhibits cell proliferation in subcutaneous PDAC tumors.

(**A**) Representative immunofluorescence staining images (left) and quantitative analysis (right) of ADAM12⁺/α-SMA⁺ (left, left), ADAM12⁺/PDGFR-β⁺ (left, middle), and ADAM12⁺/CK19⁺ (left, right) co-expression on PDAC tumors from control-vaccinated mice (v-CTRL) and ADAM12-vaccinated mice (v-A12). Data were represented as the number of cells per high power field. Representative double positive cells were indicated by white arrows respectively. ($n = 7$–8 mice in v-CTRL, $n = 8$ mice in v-A12. Data were presented as mean ± SEM. Statistical test: multiple *t*-test. Scale bar 50 μm). (**B**) Representative immunofluorescence images (left) and quantitative analysis (right) of proliferating (Ki67⁺) ADAM12⁺ cells on PDAC tumors from v-CTRL mice and v-A12 mice. ($n = 7$ mice in v-CTRL, $n = 6$ mice in v-A12. Data were presented as mean ± SEM. Statistical test: unpaired two-tailed Student's *t*-test. Scale bar 50 μm). (**C**) Representative immunofluorescence staining images (left) and quantitative analysis (right) of proliferating (Ki67⁺) tumor (CK19⁺) cells on PDAC tumors from v-CTRL mice ($n = 7$) and v-A12 mice ($n = 6$). ($n = 7$ mice in v-CTRL, $n = 6$ mice in v-A12. Data were presented as mean ± SEM. Statistical test: unpaired two-tailed Student's *t*-test. Scale bar 50 μm). (**D**) Scheme of therapeutic vaccination with gemcitabine treatment on subcutaneous KP2 PDAC tumor-bearing mice. (**E**) Representative immunofluorescence images (left) and quantitative analysis (right) of apoptotic cells (cleaved caspase 3⁺) on PDAC tumors from v-CTRL mice and v-A12 mice. ($n = 5$ mice in v-CTRL, $n = 6$ mice in v-A12. Data were presented as mean ± SEM. Statistical test: unpaired two-tailed Student's *t*-test. Scale bar 50 μm).

 © The Author(s)

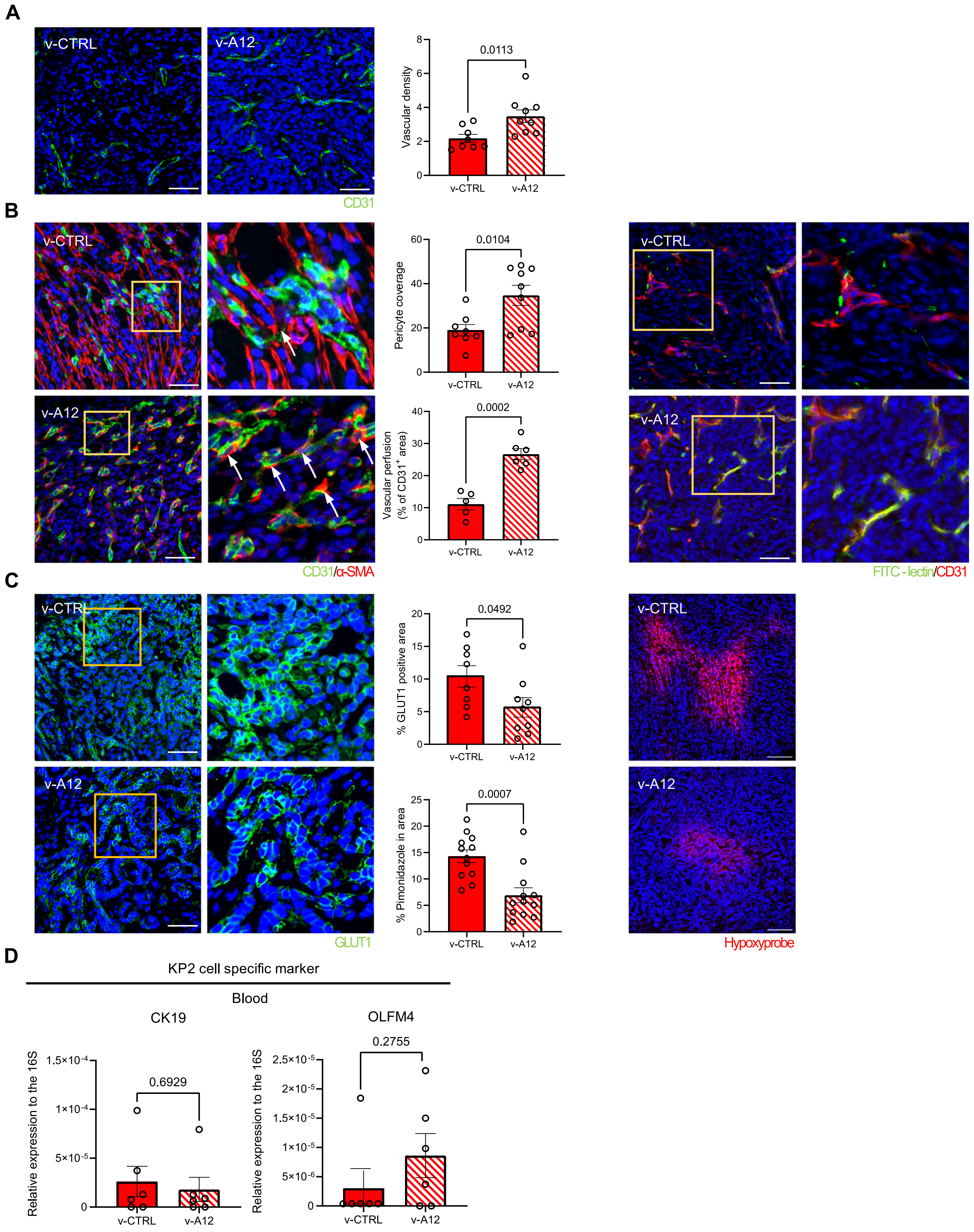
A
v-CTRL
v-A12
CD31
Vascular density
0.0113
v-CTRL
v-A12
B
v-CTRL
v-A12
CD31/α-SMA
Pericyte coverage
0.0104
Vascular perfusion (% of CD31+ area)
0.0002
v-CTRL
v-A12
FITC-lectin/CD31
C
v-CTRL
v-A12
GLUT1
% GLUT1 positive area
0.0492
% Pimonidazole in area
0.0007
Hypoxyprobe
D
KP2 cell specific marker
Blood
CK19
OLFM4
Relative expression to the 16S
0.6929
0.2755
v-CTRL
v-A12

© The Author(s)

Figure EV4. Therapeutic ADAM12 vaccination increased tumor vascular density, pericyte coverage, and decreased tumor hypoxia.

(**A**) Representative images of immunofluorescence staining (left) and quantitative analysis of CD31-positive endothelial cells (right) on subcutaneous PDAC tumors treated with control vaccine (v-CTRL) and ADAM12 vaccine (v-A12) together with representative images of CD31 immunostaining. ($n = 8$ mice in v-CTRL, $n = 9$ mice in v-A12, Data were presented as mean ± SEM. Statistical test: unpaired two-tailed Student's t-test. Scale bar 100 μm). (**B**) Representative images of immunofluorescence staining (left, right) and quantitative analysis of pericyte coverage (middle, upper) assessed by α-SMA/CD31 co-localization (yellow) as well as vascular perfusion (middle, bottom) presented by FITC-lectin^{+}/CD31^{+} cells on PDAC tumors treated with control vaccine (v-CTRL) and ADAM12 vaccine (v-A12). Representative pericytes were indicated by white arrows respectively. (Pericyte coverage: $n = 8$ mice in v-CTRL, $n = 9$ mice in v-A12; vascular perfusion: $n = 5$ mice in v-CTRL, $n = 6$ mice in v-A12. Data were presented as mean ± SEM. Statistical test: unpaired two-tailed Student's t-test. Scale bar 50 μm). (**C**) Representative images of immunofluorescence staining (left, right) and quantitative analysis of tumor hypoxia by GLUT1-positive cells (middle, upper) as well as by the coverage of Pimonidazole positive area (middle, bottom) on PDAC tumors from mice treated with control vaccine (v-CTRL, $n = 8$) or ADAM12 vaccine (v-A12, $n = 9$). (GLUT1 staining: $n = 8$ mice in v-CTRL, $n = 9$ mice in v-A12; Pimonidazole staining (hypoxyprobe): $n = 12$ mice in v-CTRL, $n = 12$ in v-A12. Data were presented as mean ± SEM. Statistical test: unpaired two-tailed Student's t-test. Scale bar 100 μm). (**D**) Gene expression analysis of the KP2-specific gene CK19 and OLFM4 in peripheral blood at endpoint (day 28 post tumor inoculation) from subcutaneous PDAC mouse model with control vaccine (v-CTRL) or ADAM12 vaccine (v-A12). ($n = 5$ mice in v-CTRL, $n = 5$ mice in v-A12. Data were presented as mean ± SEM. Statistical test: unpaired two-tailed Student's t-test).

 © The Author(s)

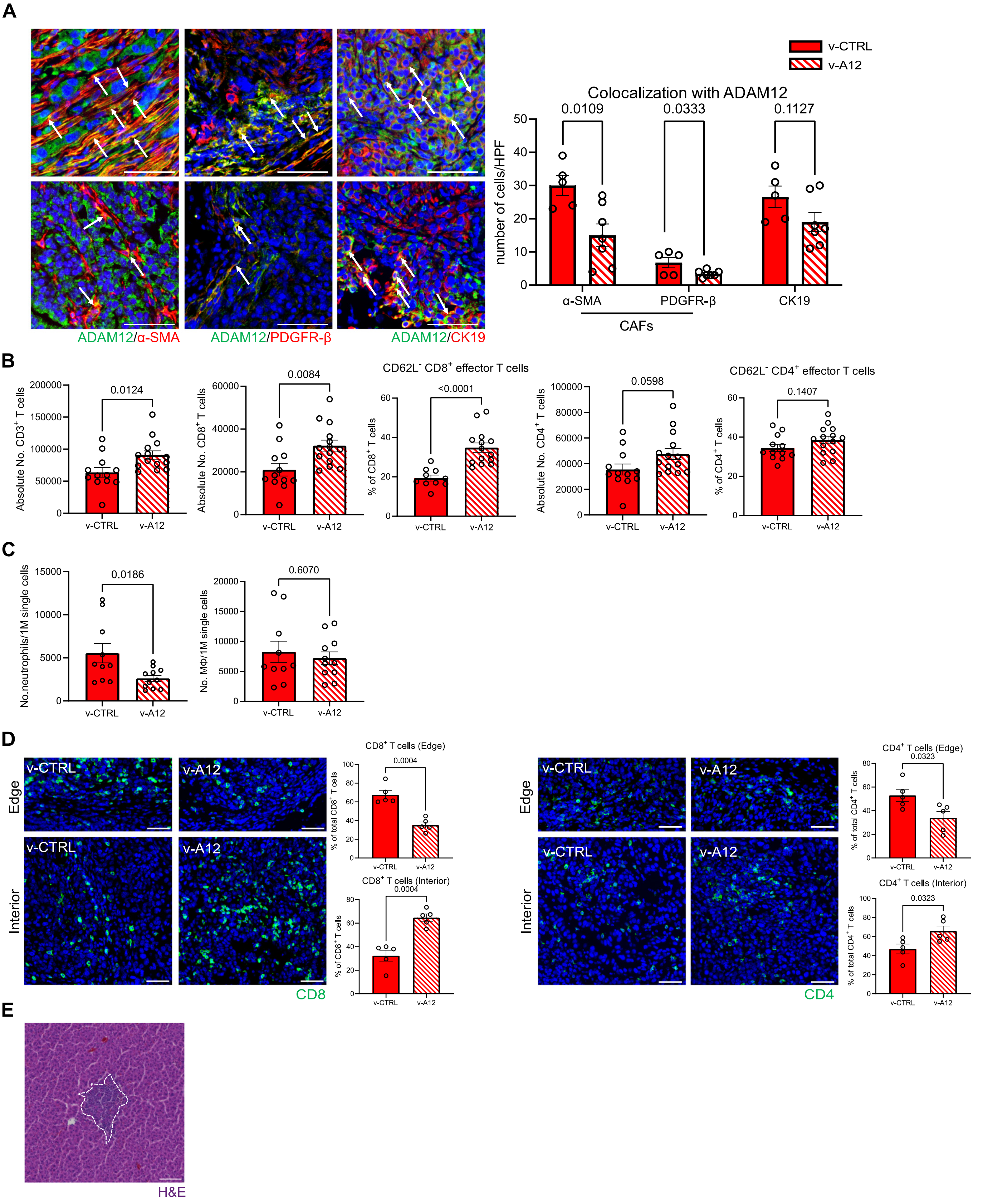
A
ADAM12/α-SMA
ADAM12/PDGFR-β
ADAM12/CK19
v-CTRL
v-A12
Colocalization with ADAM12
0.0109
0.0333
0.1127
number of cells/HPF
α-SMA
PDGFR-β
CK19
CAFs
B
Absolute No. CD3+ T cells
0.0124
Absolute No. CD8+ T cells
0.0084
CD62L- CD8+ effector T cells
% of CD8+ T cells
<0.0001
Absolute No. CD4+ T cells
0.0598
CD62L- CD4+ effector T cells
% of CD4+ T cells
0.1407
C
No.neutrophils/1M single cells
0.0186
No. MΦ/1M single cells
0.6070
D
Edge
Interior
CD8
CD8+ T cells (Edge)
0.0004
% of total CD8+ T cells
CD8+ T cells (Interior)
0.0004
% of CD8+ T cells
CD4
CD4+ T cells (Edge)
0.0323
% of total CD4+ T cells
CD4+ T cells (Interior)
0.0323
E
H&E

◀ **Figure EV5. Therapeutic ADAM12 vaccination in mice bearing orthotopic PDAC tumors decreased ADAM12^{+}CAFs, stimulated splenic CD8 T cell response, and promoted T cell relocalization within the PDAC tumor tissue.**

(**A**) Representative immunofluorescence staining images (left) and quantitative analysis (right) of ADAM12^{+}/α-SMA^{+} (left, left), ADAM12^{+}/PDGFR-β^{+} (left, middle) and ADAM12^{+}/CK19^{+} (left, right) co-expression on PDAC tumors from control-vaccinated mice (v-CTRL, $n = 5$ mice) and ADAM12-vaccinated mice (v-A12, $n = 7$ mice). Data were represented as the number of cells per high power field (HPF). Representative double positive cells were indicated by white arrows respectively. ($n = 5$ in v-CTRL, $n = 7$ in v-A12. Data were presented as mean ± SEM. Statistical test: unpaired two-tailed Student's *t*-test. Scale bar 50 μm). (**B**) Quantitative FACS analysis of the absolute number of T cells, CD8^{+} T cells, CD62L^{-}CD8^{+} effector T cells, CD4^{+} T cells, and CD62L^{-}CD4^{+} effector T cells in spleens from control-vaccinated mice (v-CTRL) and ADAM12-vaccinated mice (v-A12). ($n = 12$ mice in v-CTRL, $n = 14$ mice in v-A12. *$n = 10$ mice in v-CTRL in the CD62L^{-}CD8^{+} effector T cell population because of two outliers. Data were presented as mean ± SEM. Statistical test: unpaired two-tailed Student's *t*-test.). (**C**) Quantitative analysis of the number of neutrophils (left) and macrophages (right) per 1 M single cells in PDAC tumors from control-vaccinated mice (v-CTRL) and ADAM12-vaccinated mice (v-A12). ($n = 10$ mice in v-CTRL, $n = 11$ mice in v-A12. Data were presented as mean ± SEM. Statistical test: unpaired two-tailed Student's *t*-test.). (**D**) Representative images of CD8^{+} T cell (left) and CD4^{+} T cell (right) localization, and quantitative analysis of CD8^{+} T cell and CD4^{+} T cell population at the edge of PADC tumor tissue (right, upper) and the interior of PDAC tumor tissue (right, bottom). ($n = 5$ mice in v-CTRL, $n = 5$ mice in v-A12. Data were presented as mean ± SEM. Statistical test: unpaired two-tailed Student's *t*-test. Scale bar 50 μm). (**E**) Representative hepatic micrometastasis (area indicated within the white dotted outline) image with H&E staining, scale bar 50 μm.

 © The Author(s)
